# The murine cytomegalovirus M35 protein antagonizes type I IFN induction downstream of pattern recognition receptors by targeting NF-κB mediated transcription

**DOI:** 10.1371/journal.ppat.1006382

**Published:** 2017-05-25

**Authors:** Baca Chan, Vladimir Gonçalves Magalhães, Niels A. W. Lemmermann, Vanda Juranić Lisnić, Markus Stempel, Kendra A. Bussey, Elisa Reimer, Jürgen Podlech, Stefan Lienenklaus, Matthias J. Reddehase, Stipan Jonjić, Melanie M. Brinkmann

**Affiliations:** 1 Viral Immune Modulation Research Group, Helmholtz Centre for Infection Research (HZI), Braunschweig, Germany; 2 Institute for Virology and Research Center for Immunotherapy (FZI), University Medical Center of the Johannes Gutenberg-University Mainz, Mainz, Germany; 3 Center for Proteomics, Faculty of Medicine, University of Rijeka, Rijeka, Croatia; 4 Institute for Laboratory Animal Science, Hannover Medical School, Hannover, Germany; Blumburg Institute, UNITED STATES

## Abstract

The type I interferon (IFN) response is imperative for the establishment of the early antiviral immune response. Here we report the identification of the first type I IFN antagonist encoded by murine cytomegalovirus (MCMV) that shuts down signaling following pattern recognition receptor (PRR) sensing. Screening of an MCMV open reading frame (ORF) library identified M35 as a novel and strong negative modulator of IFNβ promoter induction following activation of both RNA and DNA cytoplasmic PRR. Additionally, M35 inhibits the proinflammatory cytokine response downstream of Toll-like receptors (TLR). Using a series of luciferase-based reporters with specific transcription factor binding sites, we determined that M35 targets NF-κB-, but not IRF-mediated, transcription. Expression of M35 upon retroviral transduction of immortalized bone marrow-derived macrophages (iBMDM) led to reduced IFNβ transcription and secretion upon activation of stimulator of IFN genes (STING)-dependent signaling. On the other hand, M35 does not antagonize interferon-stimulated gene (ISG) 56 promoter induction or ISG transcription upon exogenous stimulation of the type I IFN receptor (IFNAR). M35 is present in the viral particle and, upon MCMV infection of fibroblasts, is immediately shuttled to the nucleus where it exerts its immunomodulatory effects. Deletion of M35 from the MCMV genome and hence from the viral particle resulted in elevated type I IFN transcription and secretion *in vitro* and *in vivo*. In the absence of M35, lower viral titers are observed during acute infection of the host, and productive infection in the salivary glands was not detected. In conclusion, the M35 protein is released by MCMV immediately upon infection in order to deftly inhibit the antiviral type I IFN response by targeting NF-κB-mediated transcription. The identification of this novel viral protein reinforces the importance of timely countermeasures in the complex relationship between virus and host.

## Author summary

The herpesvirus cytomegalovirus can cause severe morbidity in immunosuppressed people and poses a much greater global problem in the context of congenital infections than the Zika virus. To establish infection, cytomegalovirus needs to modulate the antiviral immune response of its host. One of the first lines of defense against viral infections is the type I interferon response which is activated by cellular sensors called pattern recognition receptors. These receptors sense viral entry and rapidly induce the transcription of type I interferons, which are instrumental for the induction of an antiviral state in infected and surrounding cells. We have identified the first viral protein encoded by murine cytomegalovirus, the M35 protein, that counteracts type I interferon transcription downstream of multiple pattern recognition receptors. We found that this viral countermeasure occurs shortly after viral entry into the host cell, as M35 is delivered with the viral particle. M35 then localizes to the nucleus where it modulates NF-κB-mediated transcription. *In vivo*, murine cytomegalovirus deficient of the M35 protein replicates to lower levels in spleen and liver and cannot establish a productive infection in the salivary glands, which is a key site of viral transmission, highlighting the important role of M35 for the establishment of infection. Our study provides novel insights into the complex interaction between cytomegalovirus and the innate immune response of its host.

## Introduction

The ability of a virus to escape or counter host immune responses is crucial for the establishment of a successful infection in the host. Exquisite manipulation and targeting of cellular checkpoints have been hallmarks of the specific adaptation of herpesviruses to their hosts. The betaherpesvirus cytomegalovirus (CMV) encodes within its large 230 kilobases genome numerous proteins involved in the evasion of cellular innate and adaptive immune responses [1–3]. Additionally, CMV has been shown to antagonize the production of signaling molecules such as type I interferons (IFN) and proinflammatory cytokines, which are amongst the first messengers of an incoming viral attack [4].

While herpesviral infection can be asymptomatic in healthy individuals, deficiencies in innate immunity lead to severe morbidity upon infection [5]. The innate immune system is activated within hours of infection and is crucial for the initiation of the adaptive immune response. The type I IFN response is one of the first following CMV infection [6] and plays a critically protective role against murine CMV (MCMV) infection [7,8]. Additionally, human CMV (HCMV) infection can be controlled by either enhancing or inhibiting the type I IFN response in cell culture models [9–11].

Initiation of the innate immune response depends on the recognition of pathogen-associated molecular patterns (PAMP) by pattern recognition receptors (PRR). Toll-like receptors (TLR) are type I transmembrane proteins, located either at the cell surface or within endosomes, and recognize nucleic acids or structural components unique to foreign invaders [12,13]. More recently, several cytoplasmic nucleic acid sensors have been described that recognize aberrantly localized RNA or DNA. RNA sensors include RIG-I-like receptors (RLR) such as RIG-I or MDA5 [14,15]. The most recently described cytoplasmic DNA sensor (CDS) is cyclic GMP-AMP synthase (cGAS), which produces the second messenger 2’3’-cGAMP upon recognition of cytosolic DNA [16–19]. Following binding of their specific foreign ligand, TLR, RLR, and CDS recruit or activate the corresponding adaptor proteins, MyD88/TRIF, MAVS, and STING, respectively. This leads to the nuclear translocation of the transcriptional activators interferon regulatory factors (IRF) and nuclear factor κB (NF-κB), resulting in the production of the type I interferons IFNα and IFNβ, as well as of proinflammatory cytokines [20,21]. Transcription of the IFNβ gene is regulated by the concerted action of multiple transcriptional regulators [22–24]. The enhancer region in the IFNβ promoter contains binding regions, so called positive regulatory domains (PRD), for members of the AP-1 family (PRD-IV), the IRF family (PRD-III and PRD-I) and the NF-κB family (PRD-II), which all contribute to effective transcription. The subsequent binding of IFNα/β to the IFN receptor (IFNAR) leads to the upregulation of various interferon-stimulated genes (ISG), resulting in the creation of an antiviral state [25–28].

Myeloid cells are important innate immune effectors and play a crucial role in the induction of the adaptive immune response. Myeloid dendritic cells (DC) and macrophages are permissive to CMV infection [29–34] and are important vehicles for viral dissemination and serve as latency reservoirs [29,35–37]. Plasmacytoid DC (pDC) and conventional DC (cDC) are the major sources of type I IFN secretion upon encounter with MCMV [38], although the PRR signaling pathways differ. The type I IFN response in pDC is exclusively TLR-dependent, whereas RLR and CDS are important sensors in bone marrow-derived macrophages (BMDM) and cDC.

Both cytokines and receptors on myeloid cells are targeted by CMV evasion mechanisms [39,40]. Upon HCMV infection, the protein levels of the CDS interferon gamma inducible protein 16 (IFI16), as well as of the transcription factors IRF3 and NF-κB are steadily downregulated [41], indicating that viral proteins target these important determinants of CMV infection [4]. For example, HCMV pUL37x1 has been shown to antagonize signaling downstream of the RLR adaptor MAVS in HeLa cells [42] and more recently, HCMV UL83 was observed to interact with and impede oligomerization of IFI16 in the nucleus, leading to reduced IFN signaling in fibroblasts [43]. Since HCMV infection cannot be studied in the natural host and therefore the effects of HCMV immunomodulators cannot be fully understood, we chose MCMV as a model in which to dissect the intricate pathways following PRR sensing and subsequent initiation of the type I IFN response. Thus far, the only known type I IFN antagonist in MCMV is M27, which targets STAT2 for proteasomal degradation, thereby inhibiting signaling downstream of the IFNAR [6,39,44,45]. Notably, M45 is the only MCMV protein known so far to interfere with signaling downstream of PRR sensing. This anti-apoptotic protein first activates [46] and later inhibits the activation of NF-κB via interaction with the regulatory protein NF-κB essential modulator (NEMO) [47], leading to regulation of the proinflammatory cytokine response upon MCMV infection.

Here, we describe M35 as the first MCMV protein identified to indiscriminately antagonize the induction of type I IFN downstream of multiple PRR. Upon MCMV infection, M35 shuttles immediately to the nucleus to exert its immune modulatory effect by negatively regulating NF-κB-mediated transcription of type I IFN. We show that infection with an MCMV recombinant lacking M35 leads to the loss of regulation of the type I IFN response, resulting in elevated type I IFN responses and profound viral attenuation in the host.

## Results

### The MCMV M35 protein negatively modulates signaling of pattern recognition receptors

Multiple MCMV immunoevasins of natural killer cell- and T cell-mediated immunity have been identified and well characterized. Comparatively, however, the mechanisms by which MCMV shuts down innate immune signaling are poorly understood. We sought viral proteins regulating the induction of type I IFN transcription, which is the very first response upon sensing of viral nucleic acids by PRR. We rationalized that modulators of type I IFN induction encoded by MCMV would be tegument proteins, which are introduced into infected cells with the virions, or proteins expressed with immediate-early (IE) kinetics, consistent with the need to modulate the immune response immediately upon infection. To test this, we used an IFNβ-based luciferase reporter assay to screen for modulators of type I IFN transcription in MCMV. Briefly, we co-transfected NIH3T3 fibroblasts with expression constructs of untagged known or predicted tegument or IE MCMV proteins [48–50] derived from an MCMV ORF library [51] with a reporter plasmid composed of the endogenous murine IFNβ promoter upstream of the firefly luciferase gene (IFNβ-luc) as well as a Renilla luciferase construct (pRL-TK) as a transfection control. 24 hours post transfection cells were infected with Newcastle disease virus (NDV), which is sensed by RIG-I and leads to strong induction of type I IFN transcription [52].

**Fig 1:**
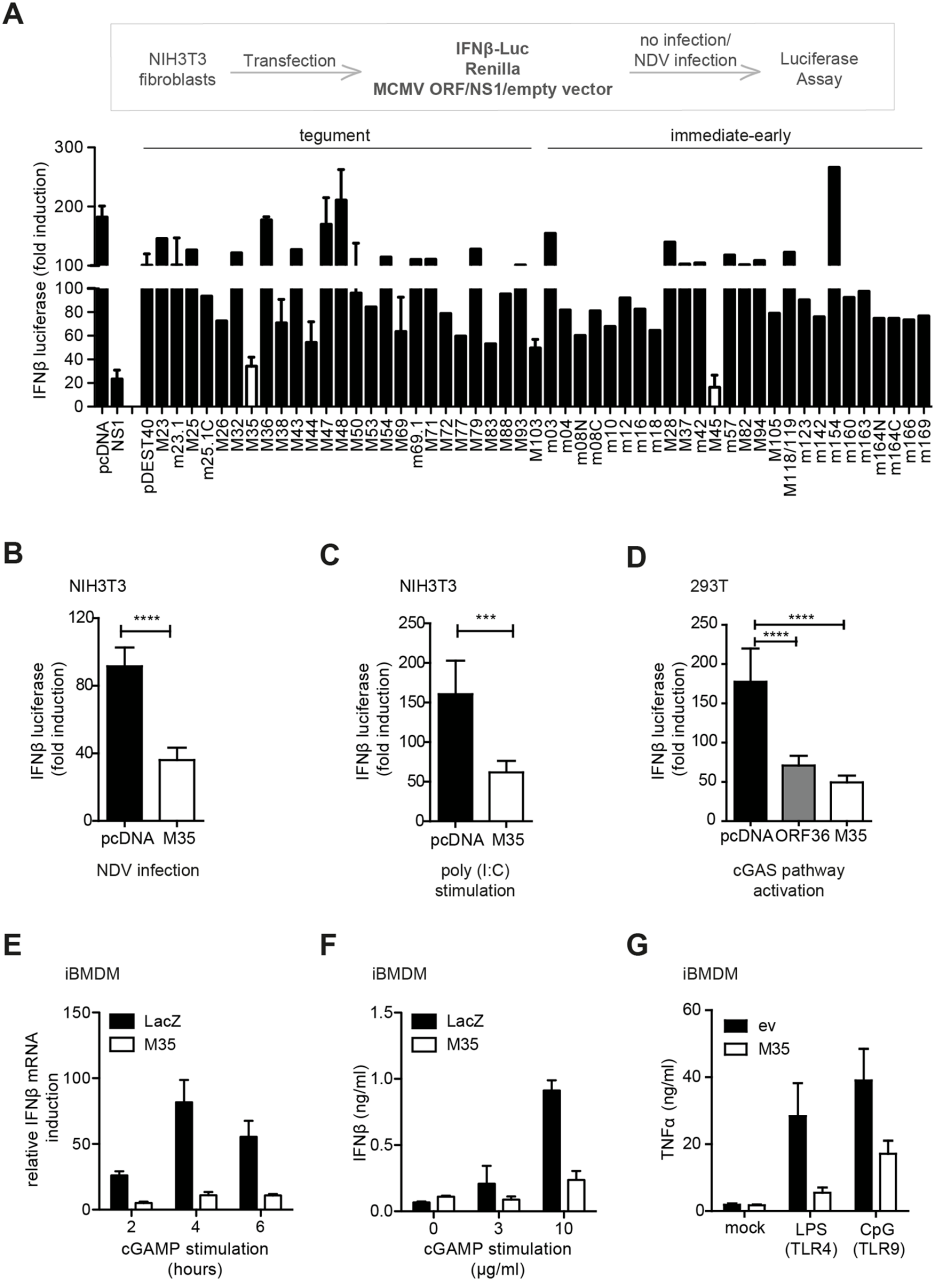
The MCMV M35 protein inhibits signaling of multiple pattern recognition receptors. (A) NIH3T3 fibroblasts were co-transfected with a reporter plasmid containing firefly luciferase under the control of the murine IFNβ promoter (IFNβ-Luc) together with a Renilla luciferase normalization control (pRL-TK) and influenza NS1 or MCMV ORFs known or predicted to code for tegument or immediate-early proteins or their corresponding empty vector (pcDNA and pDEST40, respectively). At 24 hours post transfection, cells were treated with medium or stimulated by infection with Newcastle disease virus (NDV). 21 hours p.i. cells were lysed for analysis of luciferase activity. Luciferase fold induction was calculated based on firefly luciferase values normalized to Renilla luciferase from stimulated samples divided by corresponding values from unstimulated samples. Data set is combined from one to four independent experiments and represented as mean ± SD. (B) NIH3T3 fibroblasts were co-transfected with the IFNβ-Luc and pRL-TK luciferase plasmids described in (A) as well as V5-tagged M35 or pcDNA and luciferase assay was performed following stimulation as for (A). Data is combined from three independent experiments and shown as mean ± SD. (C) NIH3T3 fibroblasts were co-transfected as described in (B) and cells stimulated with 10 μg/ml of poly(I:C) in the presence of Lipofectamine 2000 or Lipofectamine 2000 alone for 6 hours before lysis for analysis by luciferase assay. Data is combined from three independent experiments and shown as mean ± SD. (D) 293T cells were co-transfected with expression plasmids for either cGAS (stimulated) or GFP (unstimulated) together with mCherry-STING, the IFNβ-Luc and pRL-TK luciferase plasmids, and either pcDNA, myc-tagged KSHV ORF36 or V5-tagged M35. At 20 hours post transfection, cells were lysed and luciferase production was analyzed. Data is combined from four independent experiments and shown as mean ± SD. (E) Immortalized BMDM stably expressing myc-tagged LacZ or M35 were stimulated by addition of 3 μg/ml cGAMP or left unstimulated. RNA was extracted at indicated time points and IFNβ induction was measured by quantitative RT-PCR and expressed as IFNβ induction normalized to the housekeeping gene Rpl8. Data is shown as mean ± SD and combined from two independent experiments. (F) Immortalized BMDM stably expressing myc-tagged LacZ or M35 were stimulated by addition of indicated amounts of cGAMP and supernatant was collected 16 hours later and analyzed by IFNβ ELISA. Data is shown as mean ± SD and representative of two independent experiments. (G) Immortalized BMDM stably expressing myc-tagged M35 or the corresponding empty vector (pMSCV) were stimulated by addition of 10 ng/ml LPS or 1 μM CpG-B 1826 and supernatant collected at 16 hours post stimulation for analysis by TNFα ELISA. Data is combined from three independent experiments and shown as mean ± SD.***p<0.001, ****p<0.0001.

As expected, infection with NDV in the presence of empty vector alone led to high IFNβ promoter induction. As a positive control, we included influenza NS1, a well-characterized antagonist of RIG-I signaling [53–56], which clearly reduced induction of the IFNβ promoter (Fig 1A). The majority of MCMV tegument and IE proteins did not affect or only mildly affected induction of the IFNβ promoter after NDV infection and in these cases, luciferase activity was comparable to that of empty vector transfected cells (Fig 1A). However, the M45 protein, known to target NF-κB-dependent signaling [46,47], and the M35 protein strongly inhibited induction of the IFNβ promoter upon NDV infection (Fig 1A). We decided to focus on the largely uncharacterized M35 protein, since it should be present immediately after infection as a component of the viral particle [48]. The addition of a C-terminal V5-tag to M35 retained its modulatory effect on the IFNβ promoter reporter, compared to the corresponding empty vector (Fig 1B). Additionally, upon stimulation with poly(I:C) following transfection, which is sensed by the RLR RIG-I/MDA5 [57,58], we likewise observed that M35 negatively regulates IFNβ promoter induction (Fig 1C).

The cGAS-STING pathway is essential for mounting a type I IFN response against various DNA viruses [59–62]. MCMV induces STING-dependent responses [63,64] and we have observed that STING is essential for type I IFN secretion upon MCMV infection of BMDM (S1 Fig). We therefore assessed the effect of M35 on cGAS-STING-dependent type I IFN induction by an IFNβ-based luciferase reporter assay. We made use of 293T cells, which do not express endogenous cGAS or STING, and overexpressed cGAS and STING to reconstitute and activate this pathway. The cells were further co-transfected with IFNβ-luc, the Renilla construct pRL-TK, and pcDNA, ORF36-myc or M35-V5. As expected, our positive control ORF36, encoded by Kaposi's sarcoma-associated herpesvirus (KSHV) and known to inhibit IRF3 activity [65], downmodulated induction of IFNβ transcription downstream of cGAS-STING signaling. In this assay, MCMV M35 suppressed cGAS-STING dependent IFNβ transcription comparably to KSHV ORF36 (Fig 1D).

Next, we examined the effect of M35 on IFNβ transcription in BMDM. Upon stimulation of immortalized BMDM (iBMDM) stably expressing myc-tagged β-galactosidase (LacZ) or M35 with the cGAS product cGAMP, we observed strong induction of IFNβ transcription in the presence of the LacZ control (Fig 1E). In contrast, in the presence of M35, IFNβ transcription was strongly inhibited. This reduction in transcription correlates with a decrease in the levels of secreted IFNβ upon cGAMP stimulation in the presence of M35 (Fig 1F).

As MyD88-dependent signaling has been shown to be crucial for control of MCMV infection [66–68], we sought to examine if the immunomodulatory role of M35 extends to TLR signaling. Upon stimulation of iBMDM stably expressing M35-myc with the TLR4 agonist LPS or the TLR9 agonist CpG-B 1826, we observed lower levels of secreted TNFα compared to the empty vector control (Fig 1G). These data show that the M35 protein alone is a negative modulator of the induction of innate immune signaling downstream of multiple PRR.

### M35 does not target IFNAR-dependent signaling

**Fig 2:**
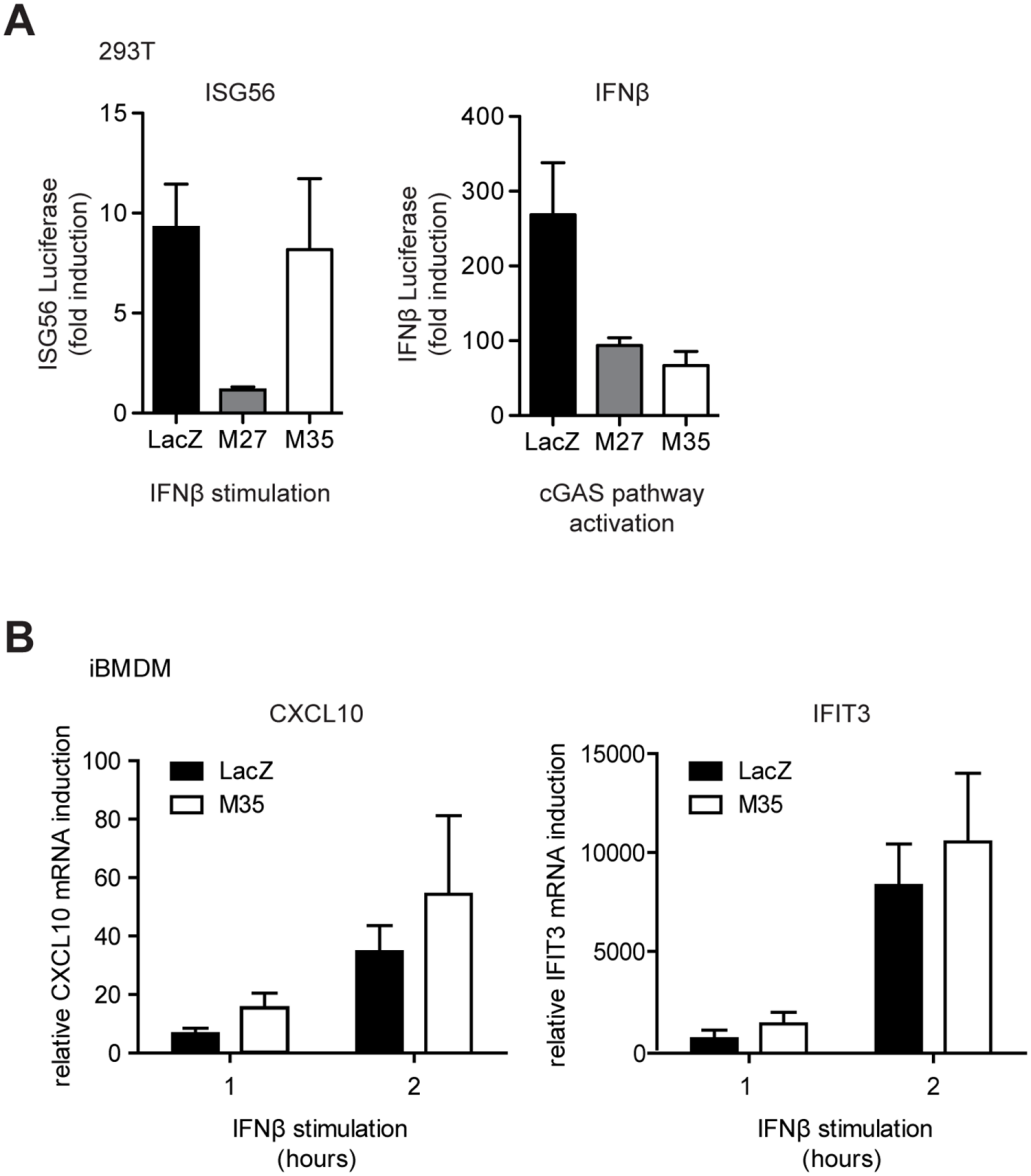
M35 does not target signaling downstream of the type I IFN receptor. (A) 293T cells were co-transfected with a reporter plasmid containing the endogenous ISG56 promoter cloned upstream of the firefly luciferase gene, pRL-TK and V5-tagged versions of LacZ, M27 or M35. 24 hours post transfection, cells were stimulated by addition of 0.1 ng/ml recombinant human IFNβ or left unstimulated. At 16 hours post stimulation, cells were lysed for analysis of luciferase production (left panel). Simultaneously, 293T cells were co-transfected with expression constructs for cGAS (stimulated) or GFP (unstimulated) together with mCherry-STING, the IFNβ-Luc and pRL-TK luciferase plasmids, and V5-tagged versions of LacZ, M27 or M35. Cells were lysed at 20 hours post transfection for analysis of luciferase production (right panel). Luciferase fold induction was calculated based on luciferase values normalized to Renilla from stimulated samples divided by corresponding values from unstimulated samples. For both panels, data is combined from four independent experiments and shown as mean ± SD. (B) Immortalized BMDM stably expressing myc-tagged LacZ or M35 were stimulated by addition of 100 U/ml of recombinant murine IFNβ or left unstimulated. Cells were lysed for RNA extraction at indicated timepoints for analysis of ISG transcription by quantitative RT-PCR (left panel: CXCL10, right panel: IFIT3) and normalized to the housekeeping gene Rpl8. Data is shown as mean ± SD and combined from three independent experiments.

To determine whether the reduced induction of IFNβ transcription observed in the presence of M35 is caused by inhibition of IFNAR signaling, which induces a positive feedback loop to further enhance IFNβ transcription, a reporter assay was utilized to assess induction of IFNAR signaling upon stimulation with recombinant IFNβ. As a positive control, we included MCMV M27, which is known to target IFNAR-dependent signaling by degrading STAT2 [44]. 293T cells were co-transfected with expression constructs of V5-tagged LacZ, M27 or M35 along with an ISG56 promoter firefly luciferase reporter plasmid and the Renilla (pRL-TK) construct. IFNAR signaling leading to ISG56 promoter induction was activated by the addition of recombinant IFNβ to the cell culture medium. Simultaneously, we performed the cGAS-STING-IFNβ luciferase based assay described in Fig 1D to verify M35 expression. As expected, M27 strongly inhibited ISG56 promoter induction upon IFNβ stimulation (Fig 2A, left panel). Interestingly, M27 also modulated IFNβ promoter activity upon activation of cGAS-STING signaling (Fig 2A, right panel). However, since 293T cells express the IFNAR [69], this result very likely reflects M27 modulation downstream of IFNAR signaling, which induces IRF7 transcription needed to further enhance IFNβ transcription. In contrast, while M35 strongly inhibited IFNβ promoter induction downstream of cGAS-STING signaling as observed earlier, it had no effect on the induction of ISG56 promoter activity upon IFNβ stimulation (Fig 2A).

In addition, we analyzed iBMDM stably expressing myc-tagged LacZ or M35 for ISG induction upon stimulation with recombinant IFNβ. While M35 inhibited IFNβ transcription upon stimulation with cGAMP (Fig 1E), we did not observe an effect on the transcription of the ISG CXCL10 and IFIT3 following stimulation with recombinant IFNβ (Fig 2B).

Taken together, these data show that M35 selectively targets transcription of IFNβ downstream of PRR, but not IFNAR-induced signal transduction upon stimulation with exogenous IFNβ.

### M35 does not affect the activation or translocation of key transcription factors

**Fig 3:**
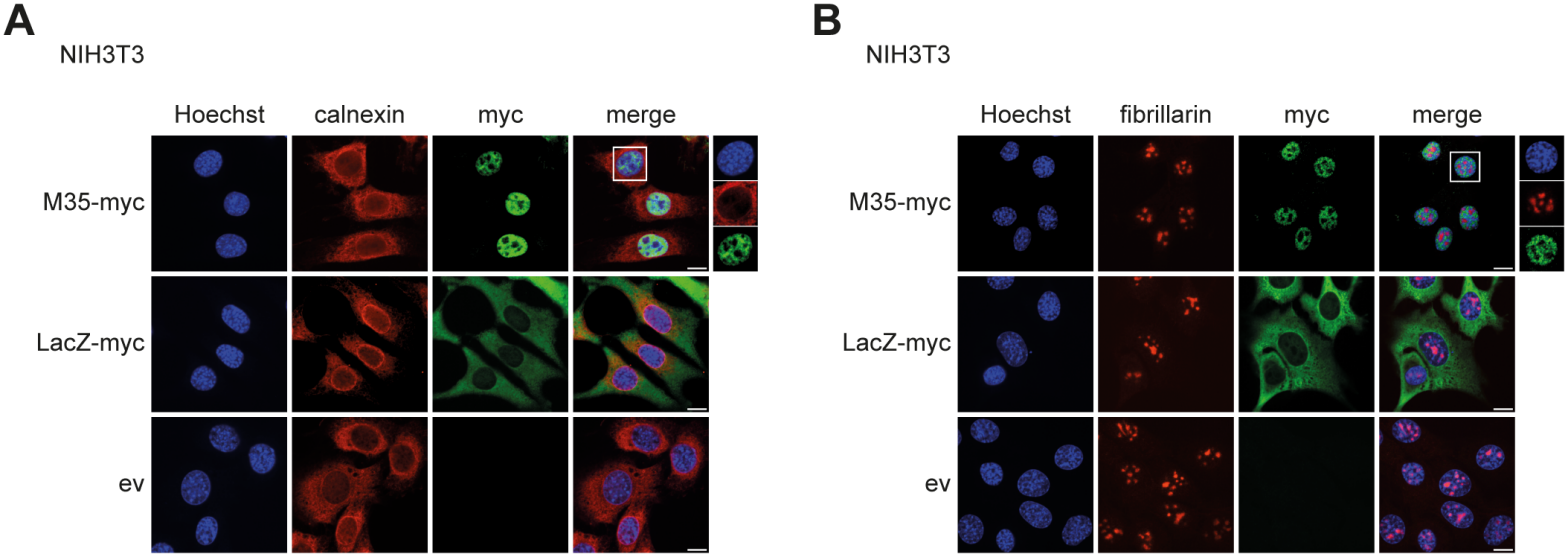
M35 is localized to the nucleus, but excluded from nucleoli. NIH3T3 fibroblasts stably expressing LacZ-myc or M35-myc or empty vector (pQCXIH) were fixed for immunolabeling with a mouse anti-myc antibody and either a rabbit anti-calnexin (A) or rabbit anti-fibrillarin (B) antibody and imaged by confocal microscopy. Nuclei were stained with Hoechst. Scale bars represent 10 μm.

**Fig 4:**
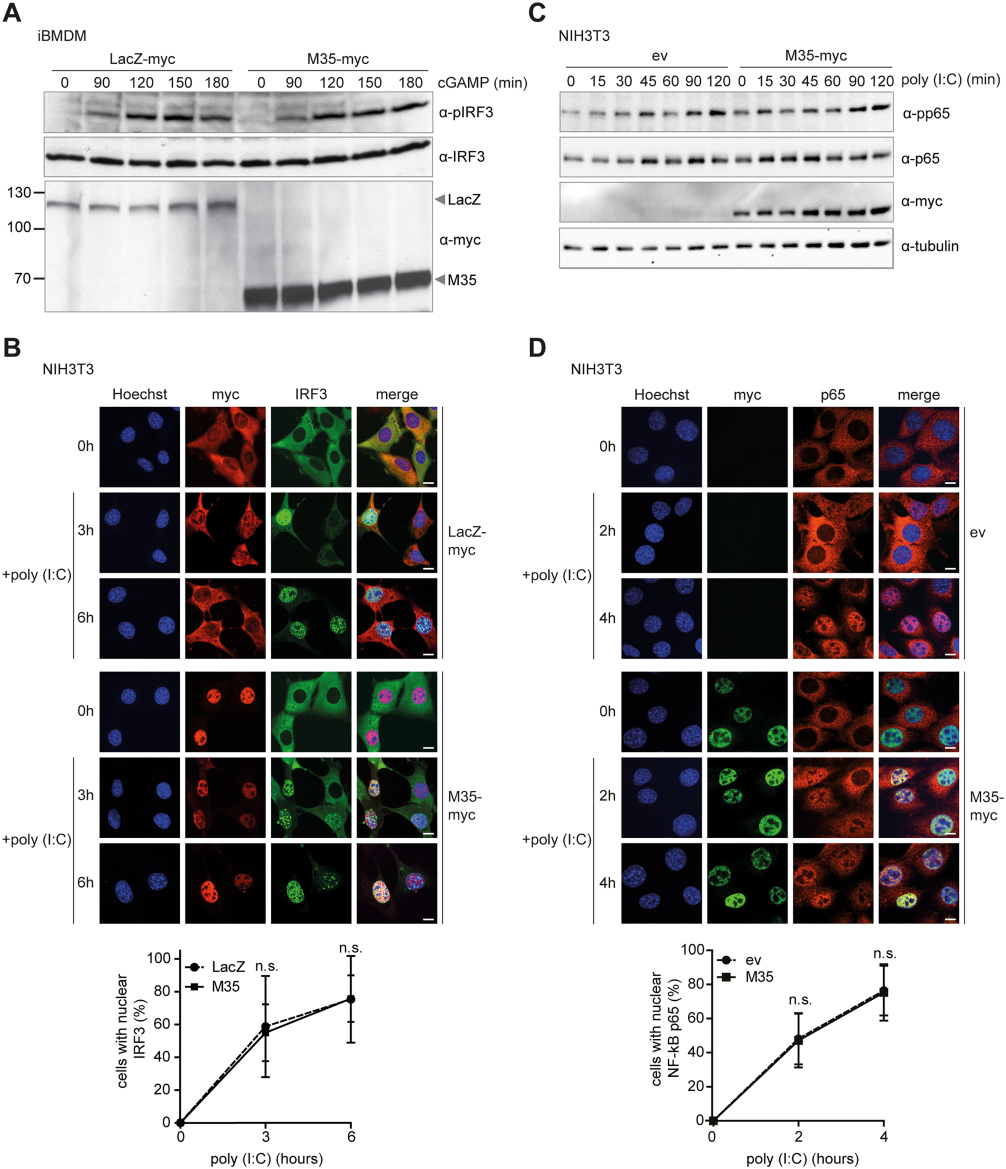
M35 does not target the phosphorylation or nuclear translocation of key transcription factors. (A) Immortalized BMDM stably expressing LacZ-myc or M35-myc were stimulated by addition of 3 μg/ml cGAMP and cells lysed at indicated time points. Lysates were separated by SDS-PAGE and endogenous IRF3 and phospho-IRF3 were detected with specific antibodies. Expression of myc-tagged LacZ and M35 was verified with a myc-specific antibody. Immunoblot shown is representative of three independent experiments. (B) NIH3T3 fibroblasts stably expressing myc-tagged LacZ or M35 and eGFP-IRF3 were stimulated by transfection of 10 μg/ml poly(I:C) with Lipofectamine 2000. At indicated times post stimulation, cells were fixed for immunolabeling with a mouse anti-myc antibody and imaged by confocal microscopy (upper panel). Nuclei were stained with Hoechst. Scale bars represent 10 μm. Corresponding counts for cells showing nuclear IRF3 (lower panel) are represented as the percentage of total cells (with a minimum of 100 cells counted per timepoint). Data is shown as mean ± SD and combined from two independent experiments. (C) NIH3T3 fibroblasts stably expressing myc-tagged M35 or corresponding empty vector pQCXIH were stimulated by transfection of 10 μg/ml poly(I:C) in the presence of Lipofectamine 2000 and cells were lysed at indicated time points. Lysates were separated by SDS-PAGE and endogenous p65, phospho-p65 and tubulin were detected with specific antibodies. Expression of myc-tagged M35 was verified with a myc-specific antibody. Immunoblot shown is representative of three independent experiments. (D) NIH3T3 fibroblasts stably expressing myc-tagged M35 or corresponding empty vector pQCXIH were stimulated as for (B). At indicated times post stimulation, cells were fixed for immunolabeling with a mouse anti-myc antibody and a rabbit anti-p65 antibody and imaged by confocal microscopy (upper panel). Nuclei were stained with Hoechst. Scale bars represent 10 μm. Corresponding counts for cells showing nuclear p65 (lower panel) are represented as percentage of total cells (with a minimum of 300 cells counted per timepoint). Data is shown as mean ± SD and combined from two independent experiments.

We have thus far shown that M35 alone is a potent inhibitor of type I IFN and proinflammatory cytokine induction downstream of multiple PRR. Next, we sought to pinpoint the stage of the innate signaling cascade which M35 targets. In NIH3T3 cells stably expressing M35-myc, we observed that M35 was not detected in the cytoplasm (Fig 3A), but rather diffusely localized in the nucleus and clearly excluded from the nucleoli (Fig 3B). This suggests that M35 likely exerts its immunomodulatory effect from the nucleus. Based on this hypothesis, phosphorylation of IRF3 and p65, which occurs in the cytoplasm upon PRR activation, as well as subsequent IRF3 and p65 nuclear translocation, should be unaffected in the presence of M35. Accordingly, we did not observe any differences in IRF3 phosphorylation (Fig 4A and S2A Fig) nor nuclear translocation upon RLR activation in the absence or presence of M35 (Fig 4B). Similarly, M35 did not affect the phosphorylation of p65 (Fig 4C and S2B Fig) nor the kinetics of p65 translocation upon RLR activation (Fig 4D).

### M35 shuts down transcription induced by the NF-κB transcription factor, but not by IRF

**Fig 5:**
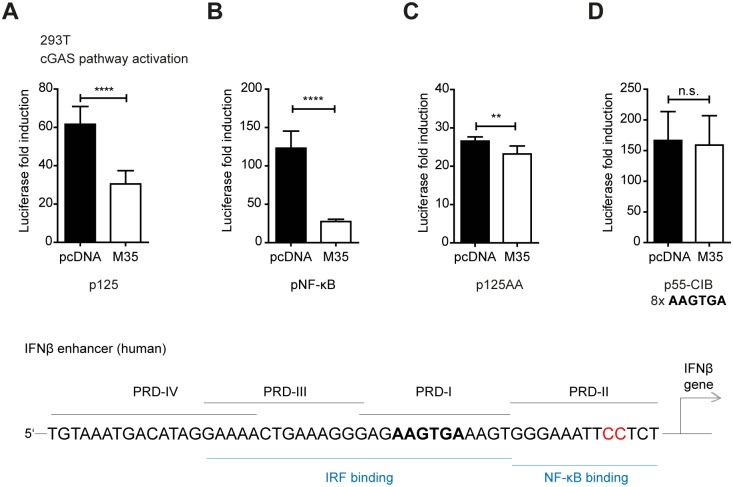
M35 targets NF-κB- but not IRF-mediated transcription. (A) 293T cells were co-transfected with expression plasmids for either cGAS (stimulated) or GFP (unstimulated) together with mCherry-STING, the p125 and pRL-TK luciferase plasmids, and V5-tagged M35 or empty vector control (pcDNA). At 20 hours post transfection, cells were lysed for analysis of luciferase production. Luciferase fold induction was calculated based on firefly luciferase values normalized to Renilla luciferase from stimulated samples divided by corresponding values from unstimulated samples. The p125 reporter contains the IFNβ enhancer consisting of PRD-IV, -III, -I and -II (sequence of the IFNβ enhancer is shown below). (B) As for (A) except with the luciferase reporter pNF-κB instead of p125. (C) As for (A) except with the p125-AA luciferase plasmid instead of p125. p125-AA contains two nucleotide exchanges within the NF-κB binding site in the PRD-II region (highlighted in red). (D) As for (A) but with the p55-CIB luciferase plasmid instead of p125. p55-CIB contains 8 tandem repeat motifs (AAGTGA, highlighted in bold in the PRD-I region), corresponding to 7 repeats of an IRF binding element. Data is combined from three (A-C) or four (D) independent experiments and shown as mean ± SD. For all, n.s. indicates not significant, **p<0.01, ****p<0.0001.

Our data has shown that M35 negatively regulates IFNβ transcription (Fig 1). Since transcription of the IFNβ gene is regulated by the concerted action of multiple transcriptional regulators and since M35 is localized to the nucleus, we sought to determine whether M35 acts by exclusively targeting IRF- or NF-κB-mediated transcription of the IFNβ gene or both. To address this, we made use of previously reported luciferase reporter plasmids: the p125 reporter consists of the human IFNβ promoter region (-125 to +19), which includes the IFNβ enhancer consisting of the PRD-IV, -III, -I and -II region (Fig 5). While IRF were shown to bind to the PRD-III and -I regions, the NF-κB transcription factor binds to the PRD-II region (Fig 5). The p125AA reporter carries two mutations (CC to AA, Fig 5) within the NF-κB binding site of the PRD-II region, which were reported to abrogate binding of NF-κB [70]. In addition, we used an NF-κB reporter containing 5 repeats of the NF-κB consensus sequence (pNF-κB). To analyze for IRF-mediated transcriptional activation, we used the p55-CIB reporter [71], which contains 8 tandem repeat motifs (AAGTGA, highlighted in bold in the PRD-I region), corresponding to seven repeats of an IRF binding element (Fig 5). We tested responsiveness of these reporters as well as of our previously described murine IFNβ reporter by activating IFNβ transcription with a constitutively active IRF3 mutant (IRF3-5D). As expected, expression of IRF3-5D resulted in activation of the IFNβ, p55-CIB, p125 and p125-AA reporters, but not of the pNF-κB reporter (S3 Fig). To analyze if M35 negatively affects transcription of these reporters, we overexpressed cGAS and STING in 293T cells to activate IFNβ transcription, and co-transfected the various reporter plasmids (Fig 5). First, we confirmed that the immunomodulatory effect of M35 was preserved upon induction of the truncated human IFNβ promoter p125, which contains the intact IFN enhancer (Fig 5A). Notably, M35 had a strong negative effect on transcription when NF-κB binding sites alone were present (pNF-κB) (Fig 5B). However, when NF-κB binding to PRD-II is disrupted by the CC to AA mutation in the PRD-II region, but IRF binding sites within PRD-III and -I are left intact (p125AA), the negative regulatory effect of M35 is strikingly less pronounced (Fig 5C). In agreement with this finding, modulation by M35 is lost if only the IRF consensus binding site is present (p55-CIB) (Fig 5D). Collectively, these data suggest that M35 negatively affects the IFNβ response downstream of PRR by targeting NF-κB-mediated, but not IRF-mediated, transcription.

### Upon MCMV infection, trafficking of tegument M35 to the nucleus precedes translocation of p65

**Fig 6:**
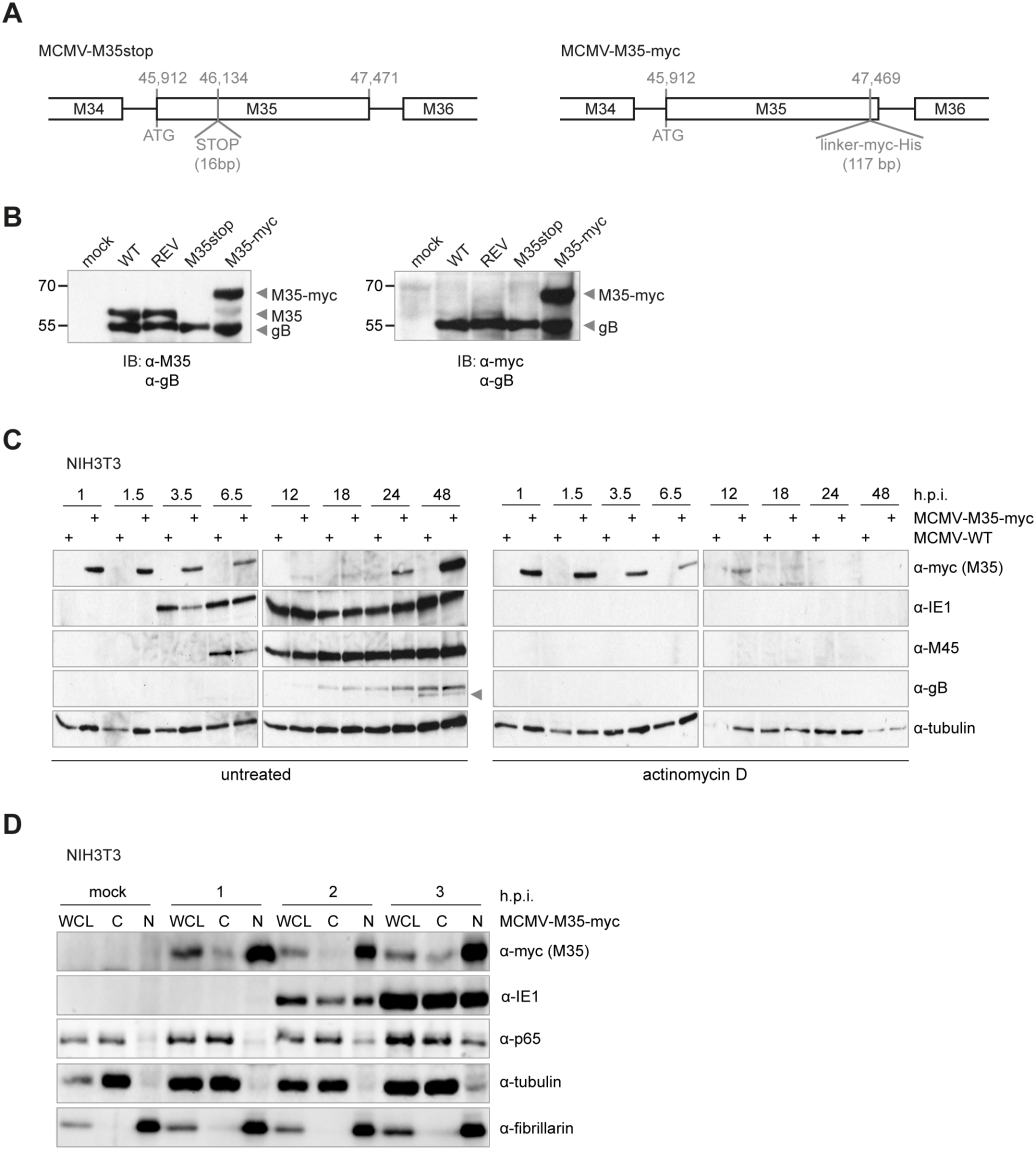
Upon MCMV infection, delivery of tegument M35 to the nucleus precedes translocation of p65. (A) Schematic representation of recombinant MCMV constructed for this study. Numbers correspond to nucleotide locations in the genome of MCMV strain Smith (accession #GU305914). Left panel: MCMV-M35stop. ATG represents the start codon of M35 and STOP denotes the introduced stop cassette. Right panel: MCMV-M35-myc. Linker-myc-His represents an 18 amino acid linker fused to a 10 amino acid myc-tag and a 6x Histidine tag. (B) Nycodenz-purified virus preparations of MCMV-WT (WT), MCMV-M35stop-REV (REV), MCMV-M35stop (M35stop) and MCMV-M35-myc (M35-myc) adjusted to 5 x 10^4^ infectious viral particles were lysed in SDS loading buffer and separated by SDS-PAGE. Mock denotes a Nycodenz-purified preparation of uninfected cells. Immunoblotting was performed with antibodies specific for M35, MCMV glycoprotein B (gB) or myc. (C) NIH3T3 fibroblasts were left untreated (left panel) or were treated with 5 μg/ml actinomycin D (right panel) 15 minutes prior to infection. MCMV-M35-myc or MCMV-WT was added at an MOI of 0.5 to the cells and infection was enhanced by centrifugation. The time point after centrifugation was defined as 0. After a 30 min incubation at 37°C to allow virus entry, unbound virus was removed with a citric acid buffer wash. Cells were then further incubated and then lysed at the indicated time points. For treatment with actinomycin D, cells were cultured in the presence of actinomycin D for the entire duration of the time course. Cell lysates were subjected to immunoblotting with antibodies specific for myc (to detect M35) as well as the MCMV proteins immediate-early protein 1 (IE1), early protein M45 or late protein gB. Tubulin levels were determined with a tubulin antibody. (D) NIH3T3 fibroblasts were either treated with media alone or infected with MCMV-M35-myc as described in (C). Whole cell lysates (WCL) were harvested at indicated time points and separated into cytoplasmic (C) and nuclear (N) fractions. Expression of myc-tagged M35 was detected by immunoblotting with an anti-myc antibody, the MCMV protein IE1 with an IE1-specific antibody, and p65 detected by an anti-p65 antibody. Tubulin and fibrillarin were used as controls for the cytoplasmic and nuclear fraction, respectively.

Next, we wanted to assess the role of M35 in evading host responses in the context of MCMV infection. We used *en passant* mutagenesis to construct several MCMV recombinants targeting M35. First, we generated a recombinant MCMV designated MCMV-M35stop, in which a 16 basepair (bp) stop cassette was inserted after the first 222 nucleotides (nt) of the M35 ORF (Fig 6A), leading to premature termination of translation of M35. In addition, we constructed a revertant virus in which expression of full-length M35 protein was restored (MCMV-M35stop-REV). Lastly, we generated a recombinant virus in which a myc/His tag was C-terminally fused to M35 (MCMV-M35-myc) (Fig 6A). To confirm the presence or absence of M35 in our recombinants, we lysed purified MCMV virions and subjected them to immunoblotting (Fig 6B). Using an M35-specific monoclonal antibody that was generated by us, we confirmed the presence of full-length M35 in both WT and MCMV-M35stop-REV virions, and the absence of M35 protein in MCMV-M35stop virions (Fig 6B). Additionally, we verified the presence of myc-tagged M35 protein in MCMV-M35-myc virions as well as low amounts of untagged M35 (Fig 6B). As a loading control, the amount of MCMV glycoprotein B (gB) was also analyzed.

Consistent with our observations, M35 has previously been shown to be virion-associated by mass spectrometry analysis [48]. However, *de novo* M35 protein expression in MCMV infected cells has not been analyzed. Reports indicate that M35 mRNA may be expressed at early or late time points in the MCMV replication cycle [50,72]. Expression and localization of virion-delivered M35 protein in the context of infection has also not yet been shown. To characterize the kinetics of M35 protein expression upon MCMV infection, we infected NIH3T3 fibroblasts with MCMV-M35-myc and analyzed M35-myc protein expression at different time points post infection by immunoblotting. The myc-specific antibody did not detect any proteins from WT MCMV infected cells demonstrating its specificity (Fig 6C). Representative immediate-early (IE1), early (M45) and late (gB) proteins were expressed with the expected kinetics. M35-myc was detected early after infection and remained stable until 6.5 hours post infection (p.i.). Little to no M35-myc was detected at 12 and 18 hours p.i., but M35 protein expression could be detected again 24 hours p.i. As expected for a viral tegument protein, we detected high levels of M35 protein at 48 hours (Fig 6C). To assess if M35 protein detected up to 6.5 hours p.i. was virion-delivered protein or *de novo* synthesized M35 protein, we performed the same expression analysis in the presence of the transcriptional inhibitor actinomycin D, which prevents any viral gene transcription. Indeed, we observed M35 protein at comparable levels to untreated cells within the first 6.5 hours of infection, but notably did not detect any M35 protein at 24 or 48 hours p.i. (Fig 6C). This indicates that M35 is delivered into infected cells as part of the virion, remains stable for several hours p.i., and is only synthesized *de novo* at late time points.

Next we wanted to investigate to which cellular compartment M35 localizes during MCMV infection at different time points p.i. We performed a cellular fractionation assay to separate the nuclear from cytoplasmic compartments of MCMV-M35-myc infected cells. To control for purity of the cellular fractions, fractions were probed with antibodies specific for tubulin (cytosolic fraction) and fibrillarin (nuclear fraction). At 1 hour p.i., M35 could be detected in the nuclear fraction (Fig 6D). This remained the case for the duration of the time course. Previous studies have reported that M35 is present at low levels in the virion [48], which likely explains our difficulties in detecting its presence in infected cells by immunofluorescence. Simultaneously, we assayed for p65 nuclear translocation, as a marker of the activation of NF-κB, in response to MCMV infection. At 1 hour p. i., p65 was restricted to the cytoplasmic fraction and only at 2 hours p. i. we could detect p65 in the nucleus in MCMV-M35-myc infected cells (Fig 6D). This suggests that the kinetics of M35 trafficking to the nucleus is more rapid than that of p65 nuclear translocation upon MCMV infection.

Collectively, these data indicate that tegument M35 is shuttled to the nucleus in a timely manner in order to counteract the onset of innate responses to MCMV infection and is only *de novo* expressed at late time points post infection.

### Tegument M35 modulates type I IFN induction in MCMV-infected macrophages

**Fig 7:**
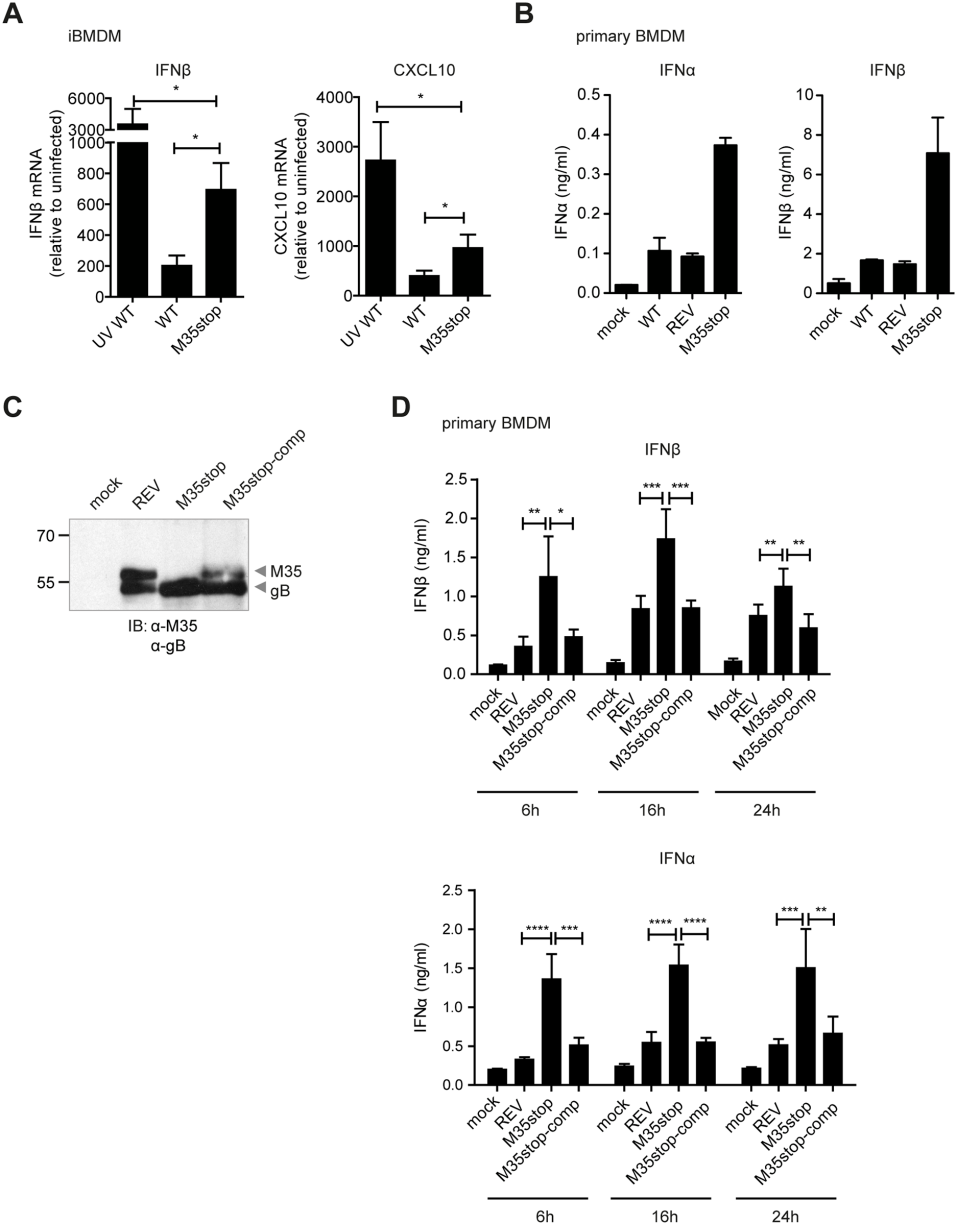
Tegument M35 modulates type I IFN responses upon MCMV infection. (A) Immortalized BMDM were infected with MCMV-WT (WT), MCMV-M35stop (M35stop) or UV-inactivated MCMV-WT (UV WT) at an MOI of 0.1 and infection was enhanced by centrifugation. Control cells were mock infected. After a 30 min incubation at 37°C followed by a citric acid buffer wash, cells were harvested for RNA extraction at 4 or 6 hours p.i. for quantification of IFNβ (left panel) and CXCL10 (right panel) transcription by quantitative RT-PCR, respectively. Values were normalized to the housekeeping gene Rpl8. Data is shown as mean ± SD and combined from three independent experiments. (B) Primary BMDM were infected with MCMV-M35stop-REV (REV) or MCMV-M35stop (M35stop) at an MOI of 0.1 or were mock infected. Supernatants were harvested 16 hours p.i. for quantification of IFNα (left panel) and IFNβ (right panel) levels by ELISA. Data is shown as mean ± SD and representative of three independent experiments. (C) Nycodenz-purified virus preparations of MCMV-WT (WT), MCMV-M35stop (M35stop) and M35-complemented MCMV-M35stop (M35stop-comp) adjusted to 5 x 10^4^ infectious viral particles were lysed in SDS-loading buffer and separated by SDS-PAGE. Mock denotes a Nycodenz-purified preparation of uninfected cells. Immunoblotting was carried out with antibodies specific for M35 and MCMV glycoprotein B (gB). (D) Primary BMDM were infected at an MOI of 0.5 by addition of MCMV-M35stop-REV (REV), MCMV-M35stop (M35stop) or M35-complemented MCMV-M35stop (M35stop-comp) to the culture medium. Supernatants were harvested at indicated timepoints p.i. for quantification of IFNβ (top panel) and IFNα (bottom panel) levels by ELISA. Data is combined from three independent experiments performed in duplicates and shown as mean ± SD. *p<0.05, **p<0.01, ***p<0.001, ****p<0.0001.

To verify that M35 modulates type I IFN induction in the context of MCMV infection, we assessed IFNβ transcription in the presence or absence of M35 in macrophages. Upon infection of iBMDM with WT MCMV, IFNβ transcription was detectable (Fig 7A). Infection with MCMV-M35stop led to an elevated induction of IFNβ transcription compared to WT MCMV. Notably, infection with UV-inactivated WT MCMV exceeded the response of MCMV-M35stop infection. Since UV treatment abrogates *de novo* expression of viral genes, this elevated IFNβ response induced by UV-inactivated MCMV indicates that M35 is not the sole antagonist of type I IFN induction in MCMV. The same trend was observed for transcription of the ISG CXCL10 (Fig 7A). We also infected primary BMDM and analyzed the levels of secreted type I IFN. We observed that secreted type I IFN levels mirrored that of transcription, in that MCMV-M35stop induced elevated levels of type I IFN compared to WT MCMV and MCMV-M35stop-REV (Fig 7B). In addition, we also observed elevated levels of IFNα in pDC and cDC infected with M35-deficient MCMV compared to MCMV-M35stop-REV (S4 Fig).

Given that the modulatory effect of M35 on type I IFN induction is apparent within the first few hours of infection, it is highly likely that tegument M35, which is delivered into the host cell by the viral particle, acts in an immunomodulatory manner. To test this hypothesis, we prepared an M35-complemented MCMV-M35stop virus stock (MCMV-M35stop-comp). The purified virus stock was generated from M2-10B4 cells stably expressing M35 infected with MCMV-M35stop, thereby incorporating M35 into the tegument, but the virus is unable to induce novel M35 protein synthesis upon infection. We confirmed successful complementation by immunoblotting with an M35-specific antibody on purified virus stocks (Fig 7C). We then infected primary BMDM and observed that infection with MCMV-M35stop-comp elicited type I IFN levels comparable to WT MCMV, whereas, as before, MCMV-M35stop induced elevated levels (Fig 7D). Notably, the restoration of the immunomodulatory effect of M35 following infection with MCMV-M35stop-comp is already apparent at 6 hours p. i., which according to the time course of M35 expression (Fig 6C) is well before the expression of newly synthesized M35. Therefore, we conclude that tegument-derived M35, but not *de novo* synthesized M35, modulates type I IFN induction (S5 Fig).

### Requirement of M35 for MCMV replication in macrophages is abolished in the absence of IFNAR

**Fig 8:**
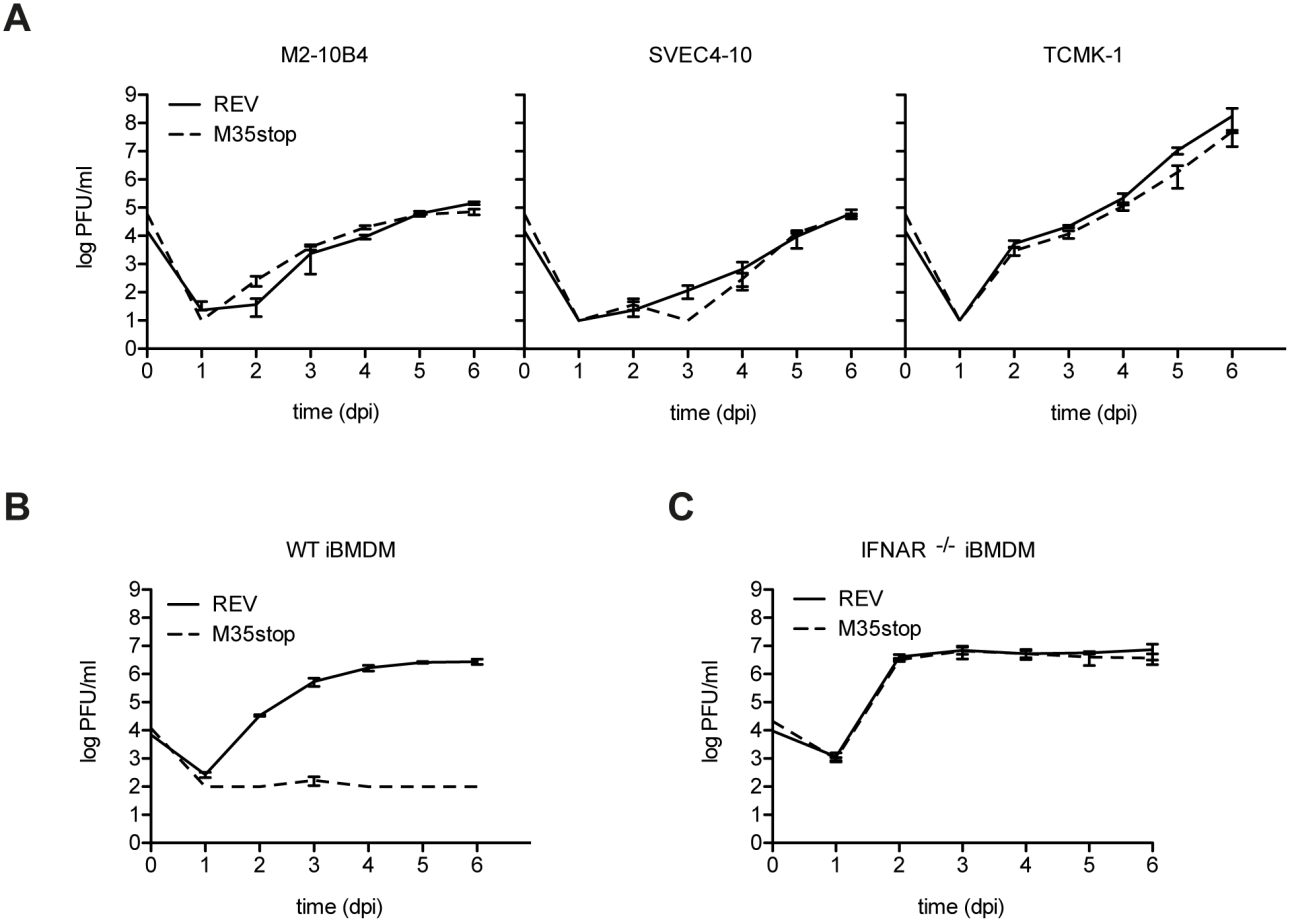
M35 is a determinant for MCMV replication in macrophages. (A) Multistep growth analysis of MCMV-M35stop-REV (REV) or MCMV-M35stop (M35stop) infection (MOI 0.05) in M2-10B4 stromal cells (left panel), SVEC-10 endothelial cells (central panel) or TCMK-1 epithelial cells (right panel). Supernatant was harvested at indicated days post infection (dpi) and viral titers were determined by standard plaque assay. The limit of detection is 10 PFU/ml. One representative of two independent experiments performed in triplicates is shown. Data is shown as mean ± SD. (B) Multistep growth analysis of MCMV-M35stop-REV (REV) or MCMV-M35stop (M35stop) infection (MOI 0.05) in immortalized WT BMDM as in (A). The limit of detection is 100 PFU/ml. One representative of two independent experiments performed in triplicates is shown. Data is shown as mean ± SD. (C) Same as for (B) but with immortalized BMDM derived from IFNAR1^-/-^ mice.

Next, we examined the role of M35 during *in vitro* MCMV replication by multistep growth curve analysis. M35 was not required for MCMV replication in cells of stromal fibroblast (M2-10B4), endothelial (SVEC4-10) or epithelial (TCMK-1) origin (Fig 8A). In contrast, deletion of M35 led to a marked growth deficit in macrophages, from which no productive infectious virus could be detected (Fig 8B). To test if the requirement of M35 for MCMV replication in macrophages could be attributed to its negative effect on type I IFN signaling, we performed growth curves in IFNAR KO iBMDM, in which the type I IFN feedback loop is effectively absent. We hypothesized that if the growth defect of MCMV lacking M35 in macrophages was due to its loss in ability to modulate type I IFN signaling, we would expect to see rescue of MCMV-M35stop in IFNAR KO iBMDM. Indeed, the ability of MCMV-M35stop to replicate in macrophages was fully restored in the absence of IFNAR-dependent signaling (Fig 8C), suggesting that the negative immunomodulatory effect of M35 is ameliorated in these cells. Our data suggest that modulation of type I IFN signaling in macrophages by M35 is crucial for the ability of MCMV to replicate in macrophages.

### M35 modulates type I IFN induction *in vivo*

**Fig 9:**
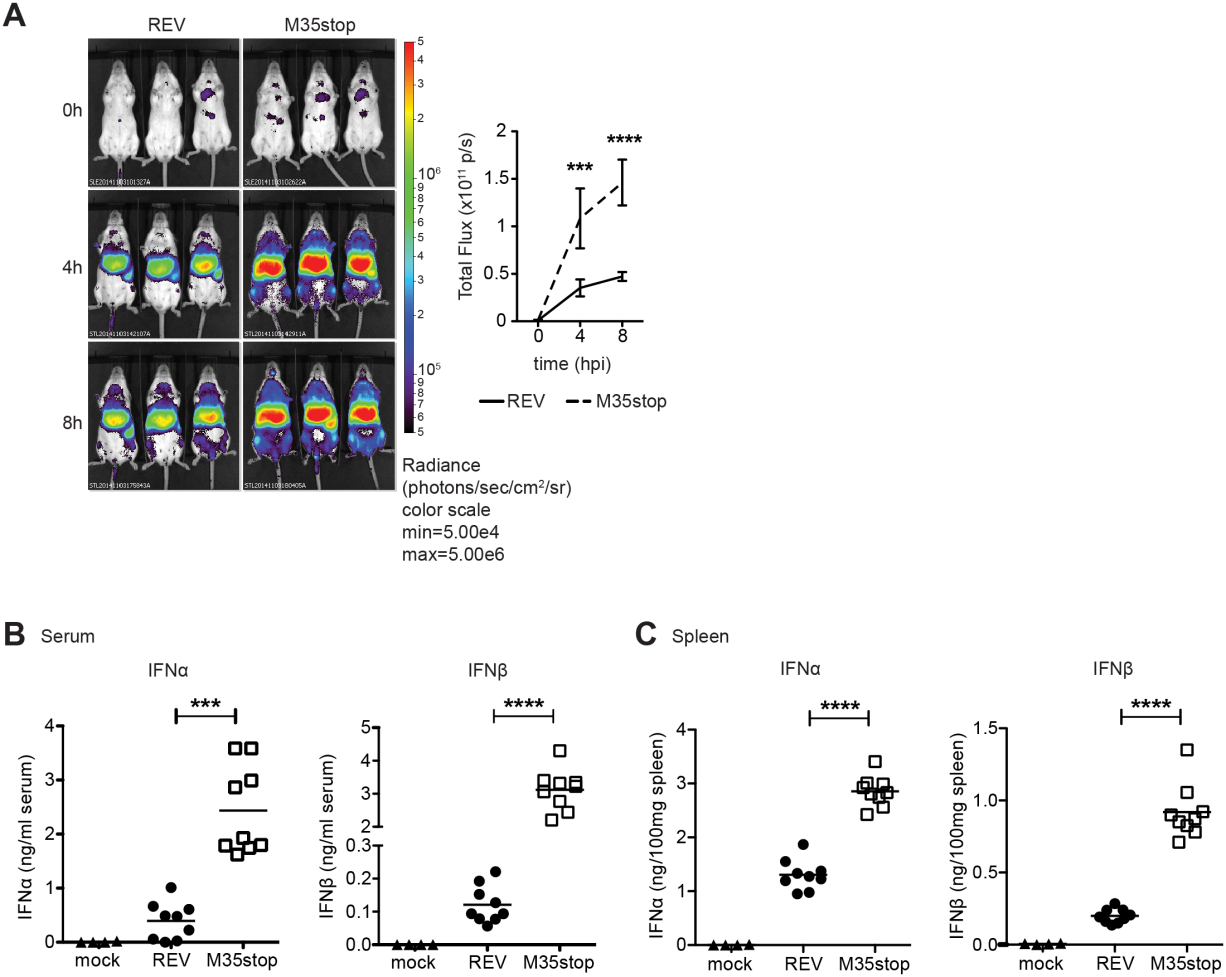
M35 inhibits the induction of type I IFN *in vivo*. (A) *In vivo* whole-body imaging of luciferase activity in IFN-β^+/Δβ-luc^ BALB/c reporter mice upon i.v. infection with 2 x 10^5^ PFU MCMV-M35stop-REV (REV) or MCMV-M35stop (M35stop) (left panel). Imaging was performed at 0, 4 and 8 hours p.i. after i.v. injection of the luciferase substrate luciferin. Right panel shows corresponding quantification of luciferase activity by region of interest analysis of the liver. Data is shown as mean ± SD and representative of two independent experiments. Type I IFN levels in serum (B) and spleen (C) following i.v. infection with 4 x 10^5^ PFU MCMV-M35stop-REV (REV) or MCMV-M35stop (M35stop) of BALB/c mice at 6 hours p.i. The levels of IFNα (respective left panel) and IFNβ (respective right panel) in the serum and spleen organ homogenates were quantified by ELISA. Mock denotes uninfected mice. Data shown is combined from two independent experiments. ***p<0.001 and ****p<0.0001.

To assess if M35 modulates type I IFN induction during MCMV infection *in vivo*, we infected transgenic mice expressing firefly luciferase under control of the natural IFNβ promoter (IFNβ-luc reporter mice) [73] with MCMV-M35stop-REV or MCMV-M35stop. We then examined the induction of IFNβ after MCMV infection by *in vivo* imaging. We observed luciferase activity in the spleen and liver at 4 and 8 hours p.i. with MCMV-M35stop-REV. Notably, compared to MCMV containing M35, luciferase activity was enhanced upon infection with MCMV-M35stop already at 4 hours and even more strongly at 8 hours p.i. (Fig 9A).

To confirm that higher levels of type I IFN were produced in response to MCMV- M35stop infection, we assayed IFNα and IFNβ levels in the serum of infected mice at 6 hours p.i. We observed significantly elevated circulating type I IFN upon infection with MCMV-M35stop (Fig 9B). A similar trend was observed in the spleen (Fig 9C). These results confirm that M35 is required for early modulation of the type I IFN response upon MCMV infection of the host.

### MCMV lacking M35 has a severe growth defect *in vivo*

**Fig 10:**
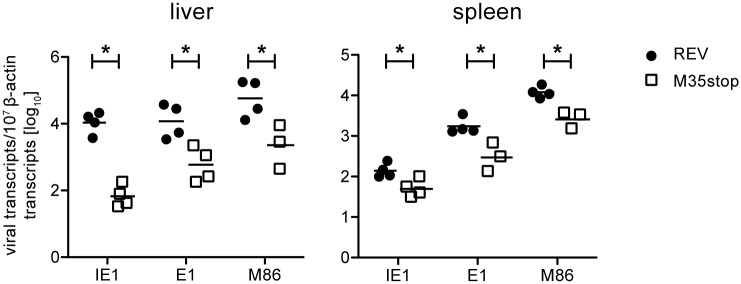
Infection with MCMV lacking M35 leads to lower levels of viral transcripts *in vivo*. BALB/c mice were infected i.v. with 2 x 10^5^ PFU MCMV-M35stop-REV (REV, black circle) or MCMV-M35-stop (M35stop, open square). Expression of viral transcripts in liver and spleen was quantitated 24 hours p.i. by quantitative RT-PCR specific for m123/IE1, m112/E1 and M86/MCP. Data were normalized to 10^7^ cellular β-actin transcripts. *p<0.05.

**Fig 11:**
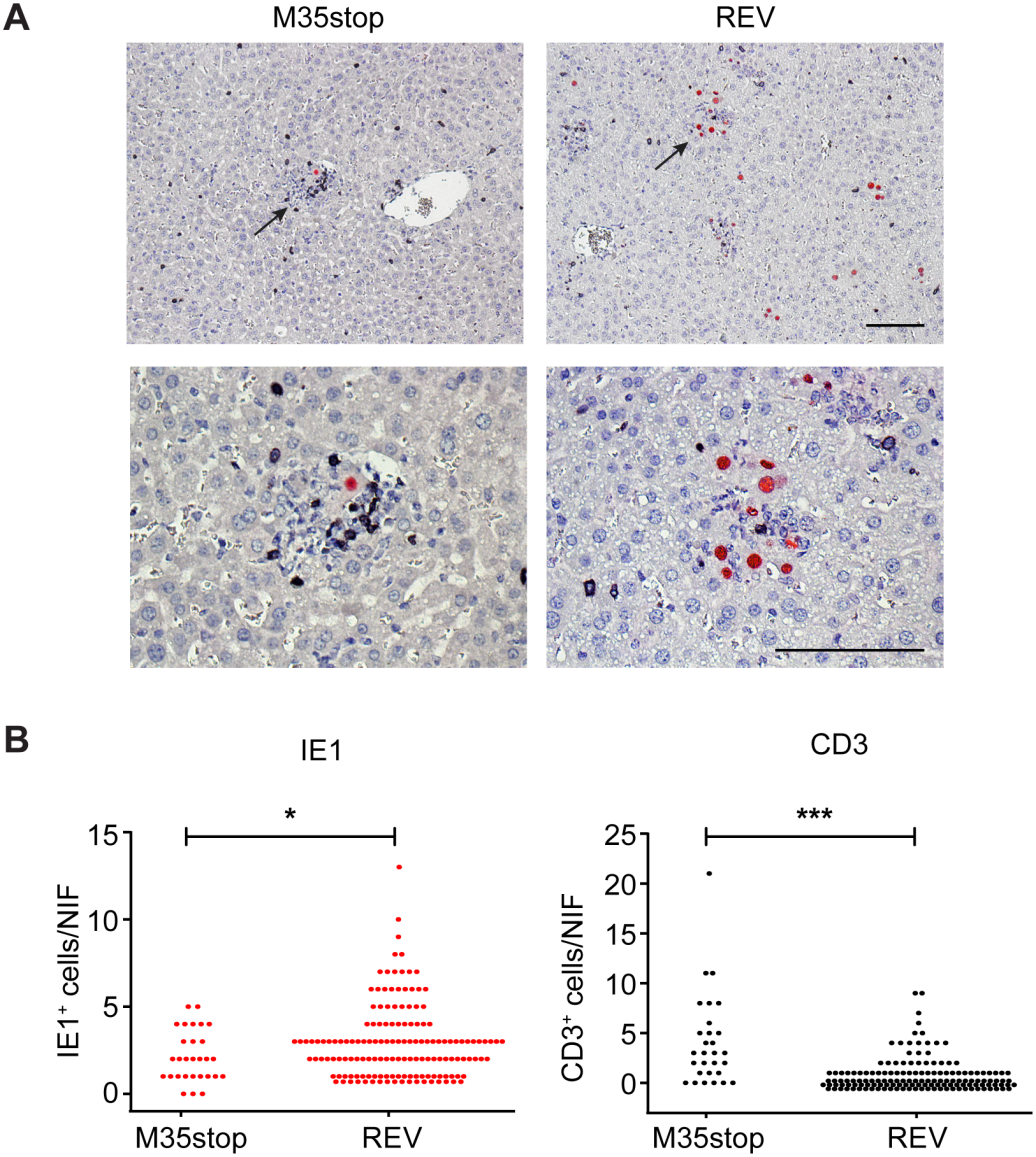
MCMV lacking M35 recruits antiviral CD3^+^ cells more efficiently to infected IE1^+^ tissue cells for the formation of protective nodular inflammatory foci (NIF). (A) For the quantification of focal infiltrates in the liver, tissue sections were collected randomly from four BALB/c mice per group on day 3 after i.v. infection with 2 x 10^5^ PFU of either MCMV-M35stop (M35stop) or MCMV-M35stop-REV (REV). Sections were stained by 2-color IHC (2C-IHC) for the expression of intranuclear viral IE1 protein (red staining) in infected liver tissue cells, as well as for the CD3ε molecule (black staining) expressed by T cells and NKT cells. Sections were counterstained with hematoxylin. Representative low-magnification overview images documenting a marked difference in the numbers of NIF (upper panels). Higher resolution images of representative foci that are marked by arrows in the overview images (lower panels). Scale bars represent 100 μm. (B) Data quantification and statistical evaluation of differences for representative tissue section areas of 40 mm^2^. Each dot symbol (n = 29 for M35stop and n = 149 for REV) represents a focus of infection or a NIF in case of CD3^+^ cell recruitment. P values were calculated by using the unpaired two-tailed Student’s t-test with Welch’s correction to account for unequal variances. Differences between data sets are considered statistically significant for *p< 0.05 and***p<0.001.

Next, we examined if modulation of type I IFN induction by M35 influences MCMV gene expression and virus replication *in vivo*. BALB/c mice were infected with MCMV-M35stop-REV or MCMV-M35stop and transcript levels for viral genes of all three temporal classes (immediate early, early and late) in the liver and spleen were analyzed 24 hours p.i. The results show that both viruses established productive infection in the tested organs, but the level of viral transcripts after MCMV-M35stop infection were significantly lower compared to MCMV-M35stop-REV infection (Fig 10). Reduced viral gene expression early after infection might result in a reduced organ manifestation at later times points. This is visible in liver sections of BALB/c mice 72 hours p.i. (Fig 11), where only very few infectious foci were visible after MCMV-M35stop infection (Fig 11A, **left panels**). In contrast, a higher number of foci were detected after infection with MCMV-M35stop-REV (Fig 11A, **right panels**). Using 2-color immunohistochemistry (2C-IHC), we simultaneously labeled the viral IE1 protein and CD3ε molecules, which are expressed by T and NKT cells. Upon infection with MCMV-M35stop we observed enhanced infiltration of CD3^+^ cells to the infected IE1^+^ liver cells (Fig 11A), thereby forming protective nodular inflammatory foci (NIF) [74–78]. After MCMV-M35stop infection, these NIF are composed of a lower number of IE1^+^ cells surrounded by a higher number of CD3^+^ cells (Fig 11B) when compared to MCMV-M35stop-REV. These data suggest improved immune control following infection with MCMV-M35stop.

**Fig 12:**
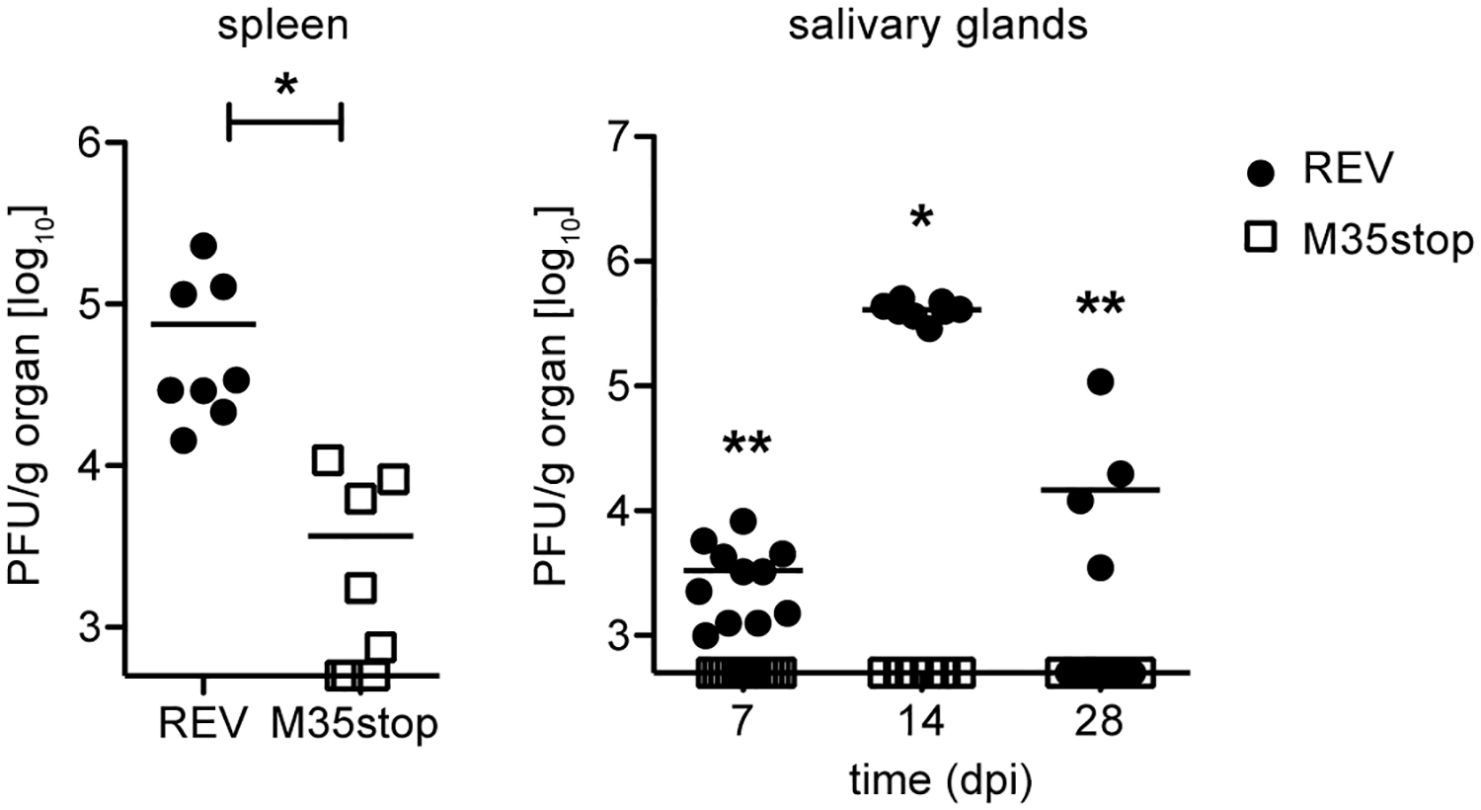
MCMV lacking M35 has a growth defect in the spleen and cannot establish chronic infection in the salivary glands. MCMV load in the spleen (left panel) and salivary glands (right panel) following i.v. infection of BALB/c mice with 2 x 10^5^ PFU MCMV-M35stop-REV (REV) or MCMV-M35stop (M35stop). Spleens were analyzed 3 days post infection (dpi) and salivary glands analyzed at indicated time points. Viral titers were determined by standard plaque assay. Data shown is combined from two independent experiments. Each symbol corresponds to an individual mouse; horizontal bars indicate mean values. *p<0.05, **p<0.01.

In parallel, viral titers in the spleen were assessed to measure viral replication during the peak phase of infection. Immunocompetent mice infected with MCMV-M35stop showed significantly reduced viral titers in the spleen 3 days p.i. compared to MCMV-M35stop-REV infected mice (Fig 12, **left panel**). A more pronounced effect was observed in the salivary glands, where MCMV-M35stop was not detectable at 7, 14, and 28 days p.i. As expected, MCMV-M35stop-REV was detectable in the salivary glands at all time points assessed, and reached high titers at day 14 p.i. (Fig 12, **right panel**). In conclusion, the lack of M35 markedly affects MCMV replication in the host at very early time points of infection, and is required for the virus to reach the salivary glands, which is the essential organ for MCMV transmission.

In summary, our study identifies M35 as a novel modulator of the type I IFN response downstream of PRR signaling and manifests its important role for viral replication in macrophages. The data also suggest that the ability of M35 to modulate the type I IFN response is crucial for MCMV replication.

## Discussion

Herpesviruses have evolved a plethora of strategies to avoid elimination by the host's immune system. To ensure the establishment of lifelong latency, herpesviruses have dedicated a large portion of their genomes to genes involved in immune modulation, which target different, and in some cases, multiple, arms of the immune system. This is well exemplified by the herpes simplex virus type 1 ICP0 protein that efficiently targets intrinsic and innate immunity [79].

Upon cell entry, viruses are faced with a variety of challenges, among them the PRR-mediated antiviral innate immune response. PRR directly bind PAMP and rapidly induce a signaling cascade leading to the transcription of type I IFN and proinflammatory cytokines. Secreted type I IFN then bind to the IFNAR, which activates a signaling cascade leading to the expression of ISG, creating an antiviral state.

In this study, we describe the identification of the poorly characterized MCMV protein, M35, as a novel negative modulator of type I IFN transcription. Our unbiased luciferase-based assay was designed on the hypothesis that MCMV must have evolved countermeasures against the induction of PRR-mediated type I IFN signaling, which is initiated within minutes of viral infection. To modulate this rapid and potent antiviral response, we postulated that either a viral protein present in the viral particle, or a viral protein with IE expression kinetics, would be a prime candidate. Our screen of known or predicted tegument and IE proteins of MCMV revealed the tegument protein M35 as a strong and novel inhibitor of IFNβ transcription. In further assays, we could confirm that M35 alone, without the aid of additional MCMV proteins, can efficiently inhibit type I IFN and proinflammatory cytokine responses downstream of multiple PRR, namely cGAS-STING, RLR and TLR. Whilst the type I IFN response is driven by both IRF- and NF-κB mediated transcription, we demonstrate that M35 targets type I IFN transcription regulated by NF-κB alone.

Deletion of M35 from the MCMV genome and hence from the viral particle results in elevated type I IFN responses *in vitro* and *in vivo*. Furthermore, we confirmed that, in the context of viral infection, M35 is a component of the viral particle and could unequivocally show for the first time that M35 is present within the infected cell for several hours after infection. Indeed, upon MCMV infection, M35 is immediately shuttled to the nucleus and precedes the translocation of p65 to the same compartment. In addition, by complementing M35-deficient MCMV with M35, we demonstrated that tegument M35 and not *de novo* synthesized M35 is responsible for downmodulation of type I IFN secretion. This is consistent with our initial hypothesis that MCMV needs to counteract the rapid PRR-mediated immune response immediately after infection.

The elevated type I IFN response in M35-deficient MCMV infected macrophages and mice leads to a strong reduction of viral growth, highlighting the indispensable role played by M35 in the causal relationship between the type I IFN response and viral growth *in vivo*. In addition, we observed that MCMV lacking M35 induces elevated IFNα secretion in pDC. Since TLR9 is the major PRR in pDC, which induces a potent type I IFN response upon MCMV infection [80], this observation suggests that M35 also modulates type I IFN transcription induced by TLR9. In BMDM, which mainly produce proinflammatory cytokines upon TLR stimulation, M35 inhibits the TLR response, resulting in reduced TNFα secretion. Since TNFα expression is regulated by NF-κB and not by IRF, these results are in line with our luciferase assays showing that M35 specifically inhibits NF-κB-, but not IRF-mediated transcription.

In a previous study, Tam and colleagues [81] have shown that a transposon insertional mutation in the MCMV M35 ORF (Smith strain) results in defective viral growth in BALB/c and severe combined immunodeficiency mice (which lack functional T and B cells), whereas *in vitro* growth in NIH3T3 fibroblasts was comparable to WT MCMV. This is consistent with our findings and underlines the importance of M35 for successful infection of its host. Moreover, we have extended upon this previous study to show that the modulation of type I IFN responses by M35 is crucial for MCMV growth.

Two lines of evidence presented in this study illustrate the necessity for MCMV to downregulate the type I IFN response in order to successfully establish an infection. First, in macrophages, which produce high levels of type I IFN, M35-deficient MCMV has a severe growth defect, whereas no growth deficit was observed in non-immune cells. Second, in the absence of the IFNAR, growth of M35-deficient MCMV was restored in macrophages. These results strongly suggest that the higher levels of type I IFN induced by M35-deficient MCMV are the cause for the severe growth defect observed in macrophages.

We have provided abundant evidence suggesting that M35 inhibits type I IFN transcription within the nucleus: (i) M35 rapidly translocates to the nucleus upon infection, (ii) M35 neither affects the phosphorylation and consequent activation of IRF3 or p65, nor the nuclear translocation of IRF3 and p65, and (iii) M35 targets NF-κB-mediated IFNβ transcription. The mechanism by which M35 translocates to the nucleus after viral entry merits further investigation, but the fact that transiently expressed M35 localizes to the nucleus suggests that the viral capsid is not necessary to direct M35 to the nuclear compartment. However, we cannot exclude the possibility that, in the presence of the viral capsid and/or associated viral and/or cellular proteins, the kinetics of M35 trafficking differ from those in cells which ectopically express M35.

The M35 sequence does not reveal homology to cellular proteins nor did analysis of its sequence reveal any motifs found in, for example, known viral transcriptional activators. The structure of the N-terminal domain of the HHV6B homologue of M35, U14, also does not give insights into its possible function [82]. Further insights into the mechanism of how M35 modulates type I IFN transcription may be obtained by the analysis of successive N-and C-terminal deletion mutants to narrow down the region important for the downmodulatory effect of M35 on type I IFN transcription. A comparative proteomics study with a nuclear M35 mutant unable to downmodulate type I IFN responses and full length M35 may reveal cellular binding partners that are either targeted or exploited by M35.

Within the human betaherpesvirus family, neither the HCMV homologue of M35, UL35, nor the U14 protein of HHV6A, HHV6B, and HHV7, have been linked to the modulation of the type I IFN response. It will be interesting to analyze if the type I IFN modulatory function is conserved among these homologues of human betaherpesviruses, despite the low degree of homology between M35 and HCMV UL35 (25.04% amino acid identity) as well as M35 and HHV6B U14 (22.02% amino acid identity). Interestingly, the HCMV tegument protein UL35 has been described to influence the cell cycle and activate the DNA damage response [83]. There is emerging evidence to suggest that DNA damage triggers the type I IFN response [84,85], which may indicate a link between the described role of UL35 in manipulating DNA damage responses and a potential role in modulating innate immune signaling.

An impressive number of viral proteins that target PRR directly or their downstream signaling pathways have been identified [86–89], and it is surprising that so far only a limited number of HCMV and MCMV proteins have been described that inhibit the PRR-mediated proinflammatory or type I IFN response. M45 blocks NF-κB activation downstream of multiple TLR as well as downstream of the interleukin 1 receptor [47]. It exerts this effect by inducing degradation of NEMO, which is the regulatory subunit of the IKK complex that acts upstream of NF-κB [47]. This function very likely explains our observation that M45 inhibits IFNβ transcription in our luciferase screen, thereby validating our screening assay. The MCMV M27 protein targets signaling directly downstream of the IFNAR [39], whereas M35 does not directly affect this pathway, demonstrating its specificity for signaling events downstream of PRR.

Our observation that UV-inactivated MCMV induces much higher type I IFN responses than M35-deficient MCMV highlights the existence of multiple MCMV-encoded modulators of the type I IFN response. It will be interesting to generate MCMV mutants that lack multiple evasion genes such as M45, M27 and M35 and different combinations thereof and compare their potential to induce the type I IFN response and establish infection. It is feasible that MCMV targets the type I IFN response at multiple steps as well as at multiple time points of the viral life cycle.

In summary, our study identifies M35 as a novel type I IFN modulator. This is the first described MCMV-encoded antagonist that interferes downstream of PRR signaling and which is crucial for MCMV to establish infection in its host. Our finding provides a solid basis for further studies on the detailed mechanism of how M35 may modulate transcription of type I IFN. This study clearly emphasizes the utmost importance of timely countermeasures by MCMV in its arms race with the host.

## Materials and methods

### Ethics statement

All animal experiments were performed in compliance with the German animal protection law (TierSchG BGBI S. 1105; 25.05.1998). The mice were handled in accordance with good animal practice as defined by FELASA and GV-SOLAS. All animal experiments were approved by the responsible state office (Lower Saxony State Office of Consumer Protection and Food Safety) under permit number #33.9-42502-04-12/0930 or by the ethics committee of the Landesuntersuchungsamt Rheinland-Pfalz, permit number 23177-07/G11-1-004.

### Mice

Mice were bred at the animal facility of the Helmholtz Centre for Infection Research in Braunschweig and in the Central Laboratory Animal Facilities at the University Medical Center Mainz and maintained under specific-pathogen-free conditions. STING knockout mice (MPYS^-/-^/Tmem173tm1Camb) have been described [90]. BALB/c mice were purchased from Janvier.

### Viruses

Manipulation of the MCMV genome was carried out by *en passant* mutagenesis [91] on the MCK-2 repaired MCMV BAC-plasmid [92]. Unless stated otherwise, MCMV-specific sequences are underlined and pEP-KanS [91] served as the template for PCR. For construction of the recombinant MCMV-M35stop, a linear PCR product was generated using primers M35stopEPfor: 5’- GCTAGAGGCCCTCCTGGCGGTCCGCGTCAAACACAGGCTG**GGCTAGTTAACTAGCC**ACGAAGGTCAGACAGACACTAGGATGACGACGATAAGTAGGG-3’ and M35stopEPrev: 5’- TGTAACAGATGACGGGCTCGAGTGTCTGTCTGACCTTCGT**GGCTAGTTAACTAGCC**CAGCCTGTGTTTGACGCGGACAACCAATTAACCAATTCTGATTAG-3’ to introduce a stop cassette (bold) at nucleotide position 46,134 (accession #GU305914).

To restore full-length M35, a linear PCR product was generated using primers M35stopReveEPfor: 5’- GCTAGAGGCCCTCCTGGCGGTCCGCGTCAAACACAGGCTGACGAAGGTCAGACAGACACTAGGATGACGACGATAAGTAGGG-3’ and M35stopReveEPrev: 5’- TGTAACAGATGACGGGCTCGAGTGTCTGTCTGACCTTCGTCAGCCTGTGTTTGACGCGGACAACCAATTAACCAATTCTGATTAG-3’ and the revertant virus was designated MCMV-M35stop-REV.

For generation of C-terminally myc-His-tagged M35 virus, designated MCMV-M35-myc, the EP-Kan-S cassette flanked by *Xba*I sites (bold) was amplified using primers *Xba*ImycHisEPfor: 5’- GCAGAA**TCTAGA**GGGCCCTTCGAACAAAAACTCATCTCAGAAGAGGATCTGAATATGCATACAGGATGACGACGATAAGTAGGG-3’ and *Xba*ImycHisEPfor 5’- ATGCAT**TCTAGA**CAACCAATTAACCAATTCTGATTAG-3’ and inserted into the *Xba*I site of pcDNA4/myc-His (Thermo Fisher) to generate pcDNA4/myc-His/EP-KanS. Here, template specific sequences are underlined. Subsequently, a linear PCR product was generated using the aforementioned pcDNA4/myc-His/EP-KanS construct as the template with primers M35mycHisEPfor: 5’- GACCGTACTCCACAGTCCAGCGCGGCCGCCGTCGGCACGAGAGCGAAGTGGAATTCTGCAGATATCCAGCA-3’ and M35mycHisEPrev: 5’-AAAGATATTTTTTATTTCTCTCTCTCTTTTATTACTATTTCTTTCCCTCAATGGTGATGGTGATGATGACC-3’ and inserted at nucleotide position 47,469 (accession #GU305914). Recombinant MCMV BACs were reconstituted after transfection of purified BAC DNA into M2-10B4 cells. To get high titer virus stocks a single clone of each recombinant was expanded on M2-10B4 cells and purified on a 10% Nycodenz cushion. The resulting virus pellets were resuspended in virus standard buffer (50 mM Tris-HCl [pH 7.8], 12 mM KCl, 5 mM EDTA) and stored at -70°C. To complement MCMV-M35stop with untagged M35, the MCMV-M35stop virus was expanded on M2-10B4 cells stably expressing untagged M35 and was designated MCMV-M35stop-comp. MCMV-GFP has previously been described [33].

### Plasmids

pRL-TK, expressing Renilla luciferase under control of the thymidine kinase promoter, is commercially available from Promega. pNF-κB Luc, containing five NF-κB responsive elements (TGGGGACTTTCCGC) upstream of the firefly luciferase gene, is commercially available from Agilent Technologies. pGL3basic-IFNβ-Luc (IFNβ-Luc) consists of the 812 bp murine IFNβ promoter region [93] cloned into pGL3basic (Promega) upstream of the firefly luciferase gene. The firefly luciferase reporter plasmids p125, consisting of the human IFNβ promoter region (-125 to +19), its related mutant p125AA Luc (CC to AA, -58) with disrupted NF-κB binding, and p55-CIB, containing 8 tandem repeat motifs (AAGTGA) corresponding to 7 repeats of an IRF binding element (AANNGAAA), were kindly provided by Takashi Fujita (Kyoto University, Japan) [70,71]. pGL3basic-ISG56-Luc (ISG56-Luc) was constructed by replacing the IFNβ promoter of pGL3basic-IFNβ-Luc with the 274 bp human ISG56 promoter.

IRF3-5D/CMVBL expresses a constitutively active form of human IRF-3 (S396D, S398D, S402D, S404D, S405D) and was kindly provided by John Hiscott (Istituto Pasteur-Fondazione Cenci Bolognetti, Italy). pEFBOS-murine cGAS, expressing untagged cGAS, and pEFBOS-mCherry-murine STING were a kind gift from Andrea Ablasser (Global Health Institute, Ecole Polytechnique Fédérale de Lausanne, Switzerland). pIRES2-GFP, pMSCV, and pQCXIH vectors were purchased from Clontech.

pWPIpuro-eGFP-IRF3, expressing an eGFP-human IRF3 fusion protein, was kindly provided by Marco Binder (University of Heidelberg, Germany) [94]. pcDNA4/LacZ-myc/His was purchased from Invitrogen. LacZ-myc/His was subcloned into pMSCVhygro via the *BamH*I/*Bgl*II sites to generate pMSCVhygro-LacZ-myc/His. pcDNA4-M35-myc/His expresses the full-length protein M35 (nucleotides 45,912–47,471 of accession #GU305914) fused to a C-terminal myc/6xHis tag. The fused sequence of M35-myc/His was subcloned into pMSCVhygro via the *Bgl*II/*Hpa*I sites to generate pMSCVhygro-M35-myc/His. The fused sequences of LacZ-myc/His and M35-myc/His were cloned into pQCXIH using *Pac*I/*Hind*III sites to generate pQCXIH-LacZ-myc/His and pQCXIH-M35-myc/His. Full length untagged M35 was subcloned into pMSCVpuro via the *Bgl*II/*EcoR*I sites to generate pMSCVpuro-M35. pcDNA3.1 M35-V5/His, pcDNA3.1 LacZ-V5/His and pcDNA3.1 M27-V5/His have been described previously [95]. The MCMV ORF library was kindly provided by Jürgen Haas (University of Edinburgh, Scotland) [51]. The library consists of untagged MCMV ORFs cloned into the Gateway cloning system pDONR207 entry vector (Invitrogen). For expression in mammalian cells, selected ORFs were transferred into the mammalian destination vector pDEST40 using the LR clonase enzyme mix.

pcDNA4-ORF36-myc/His coding for ORF36 of KSHV was previously described [96,97]. pPolI Cal NS, containing the entire vRNA sequence for the NS segment of the pandemic H1N1 influenza strain A/California/04/2009, was kindly provided by Toru Takimoto (University of Rochester Medical Center, Rochester, NY, USA) [98]. A splice acceptor site mutation was introduced in the Cal NS sequence as previously described for the 1918 NS segment [99] and Cal NS1 was then subcloned into pcDNA3.1(-) using specific primers.

All constructs were verified by sequencing. Primer sequences as well as sequences of all constructs are available upon request.

### Cell lines

M2-10B4 (ATCC CRL-1972), SVEC4-10 (ATCC CRL-2181), TCMK-1 (ATCC CCL-139) and human embryonic kidney 293-T/17 (293T, ATCC CRL-11268) cells were maintained in Dulbecco’s modified Eagle’s medium (DMEM; high glucose) supplemented with 10% fetal calf serum (FCS), 2 mM Glutamine (Gln) and 1% Penicillin/Streptomycin (P/S). The wild-type immortalized murine bone marrow-derived macrophage (iBMDM) cell line was obtained through BEI Resources, NIAID NIH (NR-9456) and cultured in the same medium as described above supplemented with 50 μM β-mercaptoethanol. The IFNAR1^-/-^ iBMDM were immortalized by Dominic De Nardo (Walter and Eliza Hall Institute of Medical Research, Parkville, VIC, Australia) from bone marrow provided by Paul J. Herzog (Hudson Institute of Medical Research, Clayton, VIC, Australia) [100]. iBMDM stably expressing LacZ-myc and M35-myc were generated by retroviral transduction using the constructs pMSCVhygro-LacZ-myc/His and pMSCVhygro-M35-myc/His and selected with 150 μg/ml hygromycin.

NIH3T3 fibroblasts (DSMZ #ACC 59) were cultured in DMEM (high glucose) supplemented with 10% FCS, 2 mM Gln, 1% P/S, 1% non-essential amino acids and 1 mM sodium pyruvate. NIH3T3 fibroblasts stably expressing LacZ-myc and M35-myc were generated by retroviral transduction using the constructs pQCXIH-LacZ-myc/His and pQCXIH-M35-myc/His and were selected with 300 μg/ml hygromycin. Control NIH3T3 cells were generated using the corresponding pQCXIH empty vector. Stable introduction of eGFP-IRF3 into NIH3T3 stably expressing LacZ-myc/His or M35-myc/His was performed by lentiviral transduction using pWPIpuro-eGFP-IRF3 and additional selection with 10 μg/ml puromycin. M2-10B4 cells stably expressing full-length untagged M35 were generated via retroviral transduction using the construct pMSCVpuro-M35 and selected with 10 μg/ml puromycin.

### Primary cells

For generation of primary plasmacytoid (pDC) and conventional dendritic cells (cDC), bone marrow was isolated from wild type C57BL/6J mice. After erythrocyte lysis, cells were cultured in RPMI 1640 medium supplemented with 10% FCS, 2 mM Gln, 1% P/S, and either 2.5% of medium from B16 cells expressing FMS-like tyrosine kinase 3 ligand (Flt3L) for pDC [101] or 20 ng/ml granulocyte macrophage colony-stimulating factor (GM-CSF, Peprotech) for cDC. On day 8, non-adherent pDC were sorted from Flt3L cultures using the pDC isolation kit II (Miltenyi Biotec) according to the manufacturer’s instructions, and non-adherent cDC were collected from GM-CSF cultures. Primary BMDM were maintained in DMEM (high glucose) supplemented with 10% FCS, 2 mM Gln, 1% P/S, 50 μM β-mercaptoethanol, and 5% macrophage colony stimulating factor (MCSF) and were prepared as described [96].

### Antibodies and reagents

Murine anti-myc-tag (#2276, clone 9B11), rabbit anti-phospho-IRF3 (#4947, clone 4D4G, Serine 396), rabbit anti-fibrillarin (#2639, clone C13C3), rabbit anti-p65 (#4764S, clone C22B4) and rabbit anti-phospho-NF-κB p65 (#3033, clone 93HI, Serine536) antibodies were purchased from Cell Signaling Technology. Rabbit anti-IRF3 (#sc-9082) was from Santa Cruz Technology. Mouse anti-tubulin (#T6199) and rabbit anti-calnexin (#C4731) were purchased from Sigma-Aldrich. Mouse monoclonal antibodies against MCMV IE1 (m123/1E1 CapRi #HR-MCMV-08), M55/gB (M55.01; HR-MCMV-05) and M45 (M45.01, CapRi #HR-MCMV-13) were generated at the Center for Proteomics (CapRi), Faculty of Medicine, University of Rijeka.

For production of mouse monoclonal antibodies directed against M35, nucleotides 4–579 (equivalent to aa 2–193) of the M35 ORF were subcloned into pET-28c (Novagen). Recombinant protein was expressed in the *E*. *coli* BL21 (DE3) bacterial strain via IPTG induction. Immunization of mice and generation of hybridoma cultures was performed as reported previously [102]. Specificity of antibodies was validated by ELISA on protein used for immunization versus irrelevant His-tagged protein, as well as on lysates of MCMV WT infected cells by immunoblotting. Antibodies were further tested on M35-myc expressing cell lysates by immunoblotting, immunoprecipitation and immunofluorescence. Selected antibodies were purified from hybridoma supernatants using protein G affinity chromatography.

2’3’-cGAMP and high molecular weight poly(I:C) were purchased from Invivogen. CpG-B 1826 and lipopolysaccharide (LPS) were purchased from MWG and Sigma-Aldrich, respectively. Lipofectamine 2000 was from Thermo Fisher Scientific and Fugene HD was purchased from Promega.

### Luciferase-based reporter assays

cGAS/STING: 293T cells (25,000/well, 96-well plate) were transiently transfected with 10 μl of Fugene HD/DNA complexes composed of 120 ng ORF expression plasmid or empty vector, 60 ng of pEFBOS-cGAS (stimulated) or pIRES2-GFP (unstimulated), 60 ng pEFBOS-mCherry-STING, 100 ng pGL3basic-IFNβ-Luc, 10 ng pRL-TK and 1.2 μl of Fugene HD (Promega) diluted in Opti-MEM (Thermo Fisher Scientific). To analyze activation of other luciferase reporters, pGL3basic-IFNβ-Luc was substituted with 50 ng p125, 50 ng p125AA, or 1 ng of p55-CIB reporter plasmids. For assaying NF-κB activity, the transfection was performed as described, except 10 ng of the pNF-κB reporter plasmid and 20 ng pRL-TK were used. pcDNA3.1(+) was added as stuffer plasmid. 20 hours post transfection cells were lysed in 1x passive lysis buffer (PLB) (Promega).

IRF3-5D: 293T cells (25,000/well, 96-well plate) were transiently transfected with 10 μl of Fugene HD/DNA complexes composed of 120 ng pcDNA3.1(+), 60 ng of IRF3-5D (stimulated) or pIRES2-GFP (unstimulated), reporter plasmid (100 ng pGL3basic-IFNβ-Luc, 50 ng p125, 50 ng p125AA, 1 ng p55-CIB, or 10 ng pNF-κB), 10 ng pRL-TK and 1 μl of Fugene HD (Promega) diluted in Opti-MEM (Thermo Fisher Scientific). pcDNA3.1(+) was added as stuffer plasmid. Cells were lysed in 1x PLB at 20 hours post transfection.

IFN/ISG56: 293T cells (25,000/well, 96-well plates) were transiently transfected with 10 μl of Fugene HD/DNA complexes composed of 120 ng ORF expression plasmid or empty vector, 100 ng pGL3basic-ISG56-Luc, 10 ng pRL-TK and 0.8 μl of Fugene HD diluted in Opti-MEM. 24 hours post transfection, cells were stimulated with 0.1 ng/ml recombinant human IFNβ (PeproTech, #300-02BC) or mock stimulated and lysed 16 hours later in 1x PLB.

RIG-I: NIH3T3 fibroblasts (50,000/well, 24-well plates) were co-transfected with 40 μl of a DNA-Fugene HD mixture consisting of 540 ng of empty vector or expression construct, 190 ng pGL3basic-IFNβ-Luc, 20 ng pRL-TK, and 2.6 μl of Fugene HD diluted in Opti-MEM. 24 hours post transfection, cells were washed with PBS and stimulated by addition of Newcastle disease virus (NDV) in Opti-MEM or treated with Opti-MEM alone. 21 hours p.i., cells were lysed in 1x PLB. For poly(I:C) stimulation, NIH3T3 cells were transfected as described above. 24 hours later, the medium was replaced with fresh medium and cells were mock-treated (Lipofectamine only) or stimulated by transfection of 5 μg poly(I:C) complexed with 5 μl Lipofectamine 2000 per well. Cells were lysed in 1x PLB 6 hours post stimulation.

Luciferase production was measured with the dual-luciferase reporter assay system and a GloMax 96-microplate luminometer (both Promega). Luciferase fold induction was calculated by dividing Renilla-normalized values from stimulated samples by corresponding values from unstimulated samples.

### *In vivo* infections and imaging

BALB/c mice were infected by the intravenous route with 4 x 10^5^ PFU of the indicated recombinant MCMVs. Virus titers in spleen and salivary glands were determined by standard plaque assay on M2-10B4 cells at indicated time points.

IFN-β^+/Δβ-luc^ reporter mice on BALB/c background (Ifnb1^tm1.2Lien^) [73] were intravenously infected with 2 x 10^5^ PFU MCMV-M35stop or MCMV-M35stop-REV for whole body *in vivo* imaging. Visualization of the reporter gene activity was achieved after intravenous injection of 150 mg/kg of D-luciferin in PBS (Calipers), followed by anesthesia using Isofluran (Baxter) and monitored by the IVIS 200 imaging system (Calipers). Photon flux was quantified using the Living Image 3.0 software. Light intensities are presented as pseudocolor overlays ranging from red (most intense) to black (least intense).

### Multistep growth curves

M2-10B4, SVEC4-10, TCMK-1 or iBMDM were infected in triplicate at an MOI of 0.05 for 1 hour at 37°C. Cells were subsequently washed with citric acid buffer (pH 3.0) to remove unbound virus and supplied with fresh culture medium. Ten percent of the supernatant was harvested and replaced every 24 hours for six days and stored at -70°C until titration on M2-10B4 cells by standard plaque assay.

### Quantitation of viral transcripts *in vivo*

Viral gene expression in liver and spleen was quantified by RT-qPCR specific for m123/IE1, M112/E1 and M86/MCP, monitoring all kinetic stages of viral replication as described previously [103]. Total RNA was isolated from organ cells using the RNeasy Mini Kit (Qiagen) according to the manufacturer’s instructions. Absolute quantification of viral transcripts was performed using graded numbers of the specific *in vitro* transcripts as standard. For normalization, cellular ß-actin transcripts were quantified in parallel.

### 2-color immunohistochemistry analysis (2C-IHC)

2C-IHC for simultaneously detecting viral IE1 protein in the nuclei of infected cells (red staining) and membrane molecule CD3ε expressed by T cells and NKT cells (black staining) was performed on liver tissue sections as described in greater detail previously [75,103,104]. In brief, IE1 was labeled specifically with monoclonal antibody CROMA 101, and red staining was achieved with alkaline phosphatase-conjugated polyclonal goat anti-mouse IgG (BioRad) and the Fuchsin+ substrate-chromogen system (Dako). CD3ε was labeled specifically with a rat monoclonal antibody, clone CD3-12 (BioRad), followed by black staining with biotin-conjugated polyclonal anti-rat Ig antibody (BD Biosciences) and the peroxidase-coupled avidin biotin complex (Vectastain Elite ABC Kit), using DAB as the substrate and ammonium nickel sulfate hexahydrate for color enhancement.

### Fractionation

Separation of cytoplasmic and nuclear compartments was performed as previously described [105] with minor modifications. Briefly, NIH3T3 fibroblasts were seeded at a density of 2.5 x 10^5^ cells per well of a 6-well plate. The next day, cells were incubated with Opti-MEM containing MCMV at an MOI of 0.5. Infection enhancement was performed by centrifugation at 805 x g for 30 minutes at 4°C. After centrifugation (defined as time point 0), cells were incubated for 30 minutes at 37°C and 7.5% CO_2_ to allow virus entry, then medium was removed and cells were washed with citric acid buffer (pH 3.0) for 2 minutes at RT to remove extracellular unbound virus. After replacement with normal media, cells were either collected immediately (time point 0.5 h) or further incubated. Cells were collected in tubes containing 300 μl PBS and centrifuged at 10,416 x g for 10 seconds at RT. Pellets were lysed with 300 μl ice-cold 0.1% NP-40 in PBS. 45 μl of the lysate were removed and combined with 15 μl of 4x SDS loading buffer (LB) and designated as the whole cell lysate (WCL). The remaining lysate was centrifuged at 16,873 x g for 10 seconds at RT and 45 μl of supernatant were added to 15 μl of 4x SDS LB and designated as the cytosolic fraction (C). The pellet was washed with 300 μl of 0.1% NP-40 in PBS followed by centrifugation at 16,873 x g for 10 seconds at RT. The pellet was resuspended in 30 μl of 1x SDS LB and designated as the nuclear fraction (N). The WCL and N fraction were sonicated twice for 5 seconds and boiled for 1 minute. For immunoblotting, 10 μl of the WCL and the C fraction and 5 μl of the N fraction were loaded per lane.

### Immunoblotting

For analysis of IRF3 and p-IRF3 protein levels, 2 x 10^6^ iBMDM stably expressing LacZ-myc or M35-myc were stimulated with 3 μg/ml 2’3’-cGAMP. At the indicated time points, cells were lysed in radioimmunoprecipitation (RIPA) buffer (20 mM Tris-HCl [pH 7.5], 1 mM EDTA, 100 mM NaCl, 1% Triton X-100, 0.5% sodium deoxycholate, 0.1% SDS) with protease and phosphatase inhibitors (Roche).

For analysis of p65 and phospho-p65 protein levels, 2.5 x 10^4^ NIH3T3 fibroblasts stably expressing M35-myc or its corresponding empty vector were stimulated by transfection of 10 μg/ml poly(I:C) complexed with Lipofectamine 2000. At the indicated time points, cells were lysed as stated above.

For analysis of MCMV protein expression levels, MCMV was added to 1 x 10^5^ NIH3T3 fibroblasts at an MOI of 0.5 and infection was enhanced by centrifugation at 805 x g at 4°C for 30 minutes. After centrifugation (defined as time point 0), cells were incubated at 37°C and 7.5% CO_2_ for 30 minutes followed by a citric acid buffer wash. At the indicated time points, cells were lysed in RIPA lysis buffer including protease inhibitors. For analysis of M35 expression kinetics in the absence of transcription, a corresponding set of cells was cultured in the presence of 5 μg/ml actinomycin D 15 minutes prior to infection and for the duration of the infection time course.

For analysis of virion-associated proteins, Nycodenz-purified virus stocks adjusted to 5 x 10^4^ PFU were directly lysed in SDS LB and analyzed by SDS-PAGE and immunoblotting.

Cell lysates were cleared by centrifugation at 17,000 x g, separated by SDS-PAGE, transferred to nitrocellulose membranes, and probed with indicated antibodies. Secondary horseradish peroxidase (HRP) coupled antibodies were then added followed by development with Lumi-Light (Roche Applied Science) or SuperSignal West Femto (Thermo Scientific) chemiluminescence substrates and membranes were exposed to film. Films were scanned and images prepared using Adobe Photoshop CS5. Intensities of phospho-IRF3 bands were measured and normalized to total IRF3 levels and phospho-p65 levels were normalized to tubulin levels using ImageJ software analysis.

### Stimulation of immortalized BMDM

Immortalized BMDM stably expressing M35-myc or LacZ-myc were stimulated with 2’3’-cGAMP (3 or 10 μg/ml) to assess type I IFN induction. For assaying ISG induction, cells were stimulated by the addition of IFNβ (100 U/ml, PBL Assay Science). For analysis of transcription by RT-qPCR, total RNA was isolated at 2, 4, and 6 hours (for 2’3’-cGAMP) or at 1 and 2 hours (for IFNβ) post stimulation using the Qiagen RNeasy Plus Mini kit according to manufacturer’s instructions. To assess TNFα induction, immortalized BMDM stably expressing M35-myc or its corresponding empty vector were stimulated with either 10 ng/ml LPS or 1 μM CpG-B 1826 for 16 hours.

### ELISA

IFNα levels were detected using a rat anti-mouse IFNα capture antibody (PBL #22100–1) and a rabbit anti-mouse IFNα detection antibody (PBL, 32100–1). IFNβ production was detected using the PBL mouse IFNβ ELISA kit (PBL #42400–1) or the LumiKine mouse IFNβ ELISA kit (Invivogen #lumi-mifnb) according to the manufacturer’s instructions. TNFα levels were detected as described previously [96].

To determine type I IFN levels in the spleen of infected mice, spleens were homogenized in 20% w/v of PBS supplemented with protease inhibitors using the FastPrep-24 instrument (MP Biomedicals). Triton X-100 was added to the homogenate to a final concentration of 0.01% and samples were incubated on ice for 15 minutes. The homogenate was clarified via centrifugation at 14,000 x g for 10 minutes at 4°C and the supernatant analyzed by ELISA.

### Quantitative RT-PCR

Immortalized BMDM were infected with MCMV at an MOI of 0.1 and infection was enhanced by centrifugation for 30 minutes at 4°C. After centrifugation, cells were incubated at 37°C and 7.5% CO_2_ for 30 minutes, washed with citric acid buffer and further incubated. At 4 and 6 hours p.i., total RNA was extracted and purified using the RNeasy Mini Kit (Qiagen) followed by DNase treatment. 100 ng of total RNA was used per reaction. Synthesis of cDNA and quantification of gene transcripts were performed by quantitative PCR using the Superscript III One-Step RT-PCR system (Invitrogen) on a LightCycler 96 instrument (Roche). *Rlp8* served as an internal control. PCR primers and Universal probe library (UPL, Roche) probes used were as follows: Rlp8 (Rlp8for: caacagagccgttgttggt, Rlp8rev: cagcctttaagataggcttgtca, UPL probe 5); IFNβ (IFNβfor: ctggcttccatcatgaacaa, IFNβrev: agagggctgtggtggagaa, UPL probe 18); CXCL10 (CXCL10for: gctgccgtcattttctgc, CXCL10rev: tctcactggcccgtcatc, UPL probe 3); IFIT3 (IFIT3for: tggactgagatttctgaactgc, IFIT3rev: agagattcccggttgacctc, UPL probe 3).

### Immunofluorescence

NIH3T3 fibroblasts stably expressing M35-myc/His, LacZ-myc/His, or corresponding empty vector were seeded onto 24-well plates on acid-washed coverslips. The next day, NIH3T3 fibroblasts were permeabilized with ice-cold methanol for 5 minutes at -20°C followed by fixation in 4% PFA in PBS for 15 minutes at RT. The coverslips were washed three times with PBS and then incubated with primary antibody diluted in 1% BSA in PBS overnight. The coverslips were washed three times with PBS and incubated with secondary antibody and Hoechst in 1% BSA in PBS for 30 minutes at RT. Coverslips were mounted on glass microscope slides with Prolong Gold (Invitrogen). Imaging was performed on a Nikon ECLIPSE Ti-E inverted microscope equipped with a spinning disk device (Perkin Elmer Ultraview) and images were processed using Volocity software (Improvision).

NIH3T3 fibroblasts stably expressing eGFP-IRF3 and M35-myc/His or eGFP-IRF3 and LacZ-myc/His were seeded onto glass coverslips. 24 hours later, the medium was replaced with fresh medium and cells were mock-treated or stimulated by transfection of 10 μg/ml poly(I:C) (Invivogen) complexed with Lipofectamine 2000. At 3 and 6 hours post stimulation, cells were fixed and visualized as described above. Quantification of IRF3 nuclear translocation was based on at least 30 images and 100 cells per condition.

To analyze p65 translocation, NIH3T3 fibroblasts stably expressing M35-myc/His or its corresponding empty vector were seeded onto glass coverslips and stimulated with poly(I:C) as described above. At 2 and 4 hours post stimulation, cells were fixed and visualized as described above. Quantification of p65 nuclear translocation was based on at least 20 images and 300 cells per condition.

### Statistical analysis

Differences between two data sets were evaluated by Student’s t-test (unpaired, two-tailed) after log-transformation with Welch’s correction or log-transformed Mann Whitney U-test using Graphpad Prism version 5.0 (GraphPad Software, San Diego, CA). P values <0.05 were considered statistically significant.

## Supporting information

S1 Fig STING plays a crucial role for detection of MCMV in primary BMDM. Primary BMDM generated from wildtype (WT) and STING knockout mice were infected with MCMV-GFP at MOI 0.5 or 2 or left uninfected and IFNα/β levels at 16 hours p.i. were analyzed by ELISA. Data is shown as mean ± SD of three independent experiments.
(TIF)

S2 Fig M35 does not affect IRF3 and p65 phosphorylation upon RLR stimulation. (A) Quantification of phospho-IRF3 levels relative to total IRF3 levels was performed on Fig 4A using ImageJ. (B) Quantification of phospho-p65 levels relative to total tubulin levels was performed on three independent experiments using ImageJ. One representative immunoblot is shown in Fig 4C.
(TIF)

S3 Fig Activation of NF-κB and IRF responsive luciferase reporter constructs upon expression of constitutively active IRF3. 293T cells were co-transfected with expression plasmids for either the constitutively active form of IRF3 designated IRF3-5D (stimulated) or GFP (unstimulated) together with the pRL-TK luciferase plasmid, pcDNA and the IFNβ, p55-CIB, pPRD-III/I, p125, p125AA or pNF-κB luciferase plasmids. At 20 hours post transfection, cells were lysed and luciferase production was analyzed. Luciferase fold induction was calculated based on firefly luciferase values normalized to Renilla luciferase from stimulated samples divided by corresponding values from unstimulated samples. Data set is combined from two independent experiments and represented as mean ± SD.
(TIF)

S4 Fig M35-deficient MCMV induces elevated IFNα secretion in dendritic cells compared to WT MCMV. pDC and cDC were infected with MCMV-M35stop-REV (REV) or MCMV-M35stop (M35stop) at an MOI of 0.01 (pDC), 0.1 (cDC), or left uninfected (mock). Supernatants were harvested 16 hours p.i. for quantification of IFNα levels by ELISA. Data is shown as mean ± SD and representative of three independent experiments.
(TIF)

S5 Fig M35 curtails type I IFN transcription downstream of multiple PRR. Sensing of MCMV infection by multiple PRR, including cGAS, RIG-I-like receptors (RLR), and Toll-like receptors (TLR), activates signaling cascades leading to the production of antiviral type I IFN. Upon MCMV infection, tegument M35 is rapidly transported to the nucleus in order to specifically interfere with NF-κB-mediated type I IFN transcription.
(TIF)

## References

[1] Jackson SE, Mason GM, Wills MR (2011) Human cytomegalovirus immunity and immune evasion. Virus Res 157: 151–160. 10.1016/j.virusres.2010.10.031 21056604

[2] Powers C, DeFilippis V, Malouli D, Fruh K (2008) Cytomegalovirus immune evasion. Curr Top Microbiol Immunol 325: 333–359. 18637515 10.1007/978-3-540-77349-8_19

[3] Lisnic B, Lisnic VJ, Jonjic S (2015) NK cell interplay with cytomegaloviruses. Curr Opin Virol 15: 9–18. 10.1016/j.coviro.2015.07.001 26208082

[4] Brinkmann MM, Dag F, Hengel H, Messerle M, Kalinke U, et al (2015) Cytomegalovirus immune evasion of myeloid lineage cells. Med Microbiol Immunol 204: 367–382. 10.1007/s00430-015-0403-4 25776081

[5] Zhang SY, Casanova JL (2015) Inborn errors underlying herpes simplex encephalitis: From TLR3 to IRF3. J Exp Med 212: 1342–1343. 10.1084/jem.2129insight4 26304982 PMC4548049

[6] Le VT, Trilling M, Zimmermann A, Hengel H (2008) Mouse cytomegalovirus inhibits beta interferon (IFN-beta) gene expression and controls activation pathways of the IFN-beta enhanceosome. J Gen Virol 89: 1131–1141. 10.1099/vir.0.83538-0 18420790

[7] Presti RM, Pollock JL, Dal Canto AJ, O'Guin AK, Virgin HWt (1998) Interferon gamma regulates acute and latent murine cytomegalovirus infection and chronic disease of the great vessels. J Exp Med 188: 577–588. 9687534 10.1084/jem.188.3.577 PMC2212470

[8] Hoebe K, Du X, Georgel P, Janssen E, Tabeta K, et al (2003) Identification of Lps2 as a key transducer of MyD88-independent TIR signalling. Nature 424: 743–748. 10.1038/nature01889 12872135

[9] Taylor RT, Bresnahan WA (2005) Human cytomegalovirus immediate-early 2 gene expression blocks virus-induced beta interferon production. J Virol 79: 3873–3877. 10.1128/JVI.79.6.3873-3877.2005 15731283 PMC1075717

[10] Sainz B Jr., LaMarca HL, Garry RF, Morris CA (2005) Synergistic inhibition of human cytomegalovirus replication by interferon-alpha/beta and interferon-gamma. Virol J 2: 14 10.1186/1743-422X-2-14 15727684 PMC554982

[11] McSharry BP, Forbes SK, Avdic S, Randall RE, Wilkinson GWG, et al (2015) Abrogation of the interferon response promotes more efficient human cytomegalovirus replication. J Virol 89: 1479–1483. 10.1128/JVI.02988-14 25392213 PMC4300662

[12] Kawai T, Akira S (2010) The role of pattern-recognition receptors in innate immunity: update on Toll-like receptors. Nat Immunol 11: 373–384. 10.1038/ni.1863 20404851

[13] Broz P, Monack DM (2013) Newly described pattern recognition receptors team up against intracellular pathogens. Nat Rev Immunol 13: 551–565. 10.1038/nri3479 23846113

[14] Chan YK, Gack MU (2015) RIG-I-like receptor regulation in virus infection and immunity. Curr Opin Virol 12: 7–14. 10.1016/j.coviro.2015.01.004 25644461 PMC5076476

[15] Yoneyama M, Onomoto K, Jogi M, Akaboshi T, Fujita T (2015) Viral RNA detection by RIG-I-like receptors. Curr Opin Immunol 32: 48–53. 10.1016/j.coi.2014.12.012 25594890

[16] Sun L, Wu J, Du F, Chen X, Chen ZJ (2013) Cyclic GMP-AMP synthase is a cytosolic DNA sensor that activates the type I interferon pathway. Science 339: 786–791. 10.1126/science.1232458 23258413 PMC3863629

[17] Wu J, Sun L, Chen X, Du F, Shi H, et al (2013) Cyclic GMP-AMP is an endogenous second messenger in innate immune signaling by cytosolic DNA. Science 339: 826–830. 10.1126/science.1229963 23258412 PMC3855410

[18] Dempsey A, Bowie AG (2015) Innate immune recognition of DNA: A recent history. Virology 479–480: 146–152. 10.1016/j.virol.2015.03.013 25816762 PMC4424081

[19] Cai X, Chiu YH, Chen ZJ (2014) The cGAS-cGAMP-STING pathway of cytosolic DNA sensing and signaling. Mol Cell 54: 289–296. 10.1016/j.molcel.2014.03.040 24766893

[20] Luecke S, Paludan SR (2016) Molecular requirements for sensing of intracellular microbial nucleic acids by the innate immune system. Cytokine. 10.1016/j.cyto.2016.10.003 27751656

[21] Roers A, Hiller B, Hornung V (2016) Recognition of endogenous nucleic acids by the innate immune system. Immunity 44: 739–754. 10.1016/j.immuni.2016.04.002 27096317

[22] Bonnefoy E, Bandu MT, Doly J (1999) Specific binding of high-mobility-group I (HMGI) protein and histone H1 to the upstream AT-rich region of the murine beta interferon promoter: HMGI protein acts as a potential antirepressor of the promoter. Mol Cell Biol 19: 2803–2816. 10082546 10.1128/mcb.19.4.2803 PMC84073

[23] Escalante CR, Yie J, Thanos D, Aggarwal AK (1998) Structure of IRF-1 with bound DNA reveals determinants of interferon regulation. Nature 391: 103–106. 10.1038/34224 9422515

[24] Panne D, Maniatis T, Harrison SC (2007) An atomic model of the interferon-beta enhanceosome. Cell 129: 1111–1123. 10.1016/j.cell.2007.05.019 17574024 PMC2020837

[25] Schlee M, Hartmann G (2016) Discriminating self from non-self in nucleic acid sensing. Nat Rev Immunol 16: 566–580. 10.1038/nri.2016.78 27455396 PMC7097691

[26] Schoggins JW (2014) Interferon-stimulated genes: roles in viral pathogenesis. Curr Opin Virol 6: 40–46. 10.1016/j.coviro.2014.03.006 24713352 PMC4077717

[27] Hoffmann HH, Schneider WM, Rice CM (2015) Interferons and viruses: an evolutionary arms race of molecular interactions. Trends Immunol 36: 124–138. 10.1016/j.it.2015.01.004 25704559 PMC4384471

[28] Schneider WM, Chevillotte MD, Rice CM (2014) Interferon-stimulated genes: a complex web of host defenses. Annu Rev Immunol 32: 513–545. 10.1146/annurev-immunol-032713-120231 24555472 PMC4313732

[29] Reeves MB, MacAry PA, Lehner PJ, Sissons JG, Sinclair JH (2005) Latency, chromatin remodeling, and reactivation of human cytomegalovirus in the dendritic cells of healthy carriers. Proc Natl Acad Sci U S A 102: 4140–4145. 10.1073/pnas.0408994102 15738399 PMC554799

[30] Sinclair J, Reeves M (2014) The intimate relationship between human cytomegalovirus and the dendritic cell lineage. Front Microbiol 5: 389 10.3389/fmicb.2014.00389 25147545 PMC4124589

[31] Dalod M, Hamilton T, Salomon R, Salazar-Mather TP, Henry SC, et al (2003) Dendritic cell responses to early murine cytomegalovirus infection: subset functional specialization and differential regulation by interferon alpha/beta. J Exp Med 197: 885–898. 10.1084/jem.20021522 12682109 PMC2193893

[32] Riegler S, Hebart H, Einsele H, Brossart P, Jahn G, et al (2000) Monocyte-derived dendritic cells are permissive to the complete replicative cycle of human cytomegalovirus. J Gen Virol 81: 393–399. 10.1099/0022-1317-81-2-393 10644837

[33] Mathys S, Schroeder T, Ellwart J, Koszinowski UH, Messerle M, et al (2003) Dendritic cells under influence of mouse cytomegalovirus have a physiologic dual role: to initiate and to restrict T cell activation. J Infect Dis 187: 988–999. 10.1086/368094 12660946

[34] Busche A, Jirmo AC, Welten SPM, Zischke J, Noack J, et al (2013) Priming of CD8(+) T cells against cytomegalovirus-encoded antigens is dominated by cross-presentation. J Immunol 190: 2767–2777. 10.4049/jimmunol.1200966 23390296

[35] Stoddart CA, Cardin RD, Boname JM, Manning WC, Abenes GB, et al (1994) Peripheral blood mononuclear phagocytes mediate dissemination of murine cytomegalovirus. J Virol 68: 6243–6253. 8083964 10.1128/jvi.68.10.6243-6253.1994 PMC237044

[36] Stern JL, Slobedman B (2008) Human cytomegalovirus latent infection of myeloid cells directs monocyte migration by up-regulating monocyte chemotactic protein-1. J Immunol 180: 6577–6585. 18453576 10.4049/jimmunol.180.10.6577

[37] Taylor-Wiedeman J, Sissons JG, Borysiewicz LK, Sinclair JH (1991) Monocytes are a major site of persistence of human cytomegalovirus in peripheral blood mononuclear cells. J Gen Virol 72 (Pt 9): 2059–2064. 1654370 10.1099/0022-1317-72-9-2059

[38] Doring M, Lessin I, Frenz T, Spanier J, Kessler A, et al (2014) M27 expressed by cytomegalovirus counteracts effective type I interferon induction of myeloid cells but not of plasmacytoid dendritic cells. J Virol 88: 13638–13650. 10.1128/JVI.00216-14 25231302 PMC4248974

[39] Zimmermann A, Trilling M, Wagner M, Wilborn M, Bubic I, et al (2005) A cytomegaloviral protein reveals a dual role for STAT2 in IFN-{gamma} signaling and antiviral responses. J Exp Med 201: 1543–1553. 10.1084/jem.20041401 15883169 PMC2212917

[40] Loewendorf A, Kruger C, Borst EM, Wagner M, Just U, et al (2004) Identification of a mouse cytomegalovirus gene selectively targeting CD86 expression on antigen-presenting cells. J Virol 78: 13062–13071. 10.1128/JVI.78.23.13062-13071.2004 15542658 PMC524971

[41] Weekes MP, Tomasec P, Huttlin EL, Fielding CA, Nusinow D, et al (2014) Quantitative temporal viromics: an approach to investigate host-pathogen interaction. Cell 157: 1460–1472. 10.1016/j.cell.2014.04.028 24906157 PMC4048463

[42] Castanier C, Garcin D, Vazquez A, Arnoult D (2010) Mitochondrial dynamics regulate the RIG-I-like receptor antiviral pathway. EMBO Rep 11: 133–138. 10.1038/embor.2009.258 20019757 PMC2828750

[43] Li T, Chen J, Cristea IM (2013) Human cytomegalovirus tegument protein pUL83 inhibits IFI16-mediated DNA sensing for immune evasion. Cell Host Microbe 14: 591–599. 10.1016/j.chom.2013.10.007 24237704 PMC3876934

[44] Trilling M, Le VTK, Fiedler M, Zimmermann A, Bleifuß E, et al (2011) Identification of DNA-damage dna-binding protein 1 as a conditional essential factor for cytomegalovirus replication in interferon-γ-stimulated cells. PLoS Pathogens 7: e1002069 10.1371/journal.ppat.1002069 21698215 PMC3116810

[45] Trilling M, Le VT, Rashidi-Alavijeh J, Katschinski B, Scheller J, et al (2014) "Activated" STAT proteins: a paradoxical consequence of inhibited JAK-STAT signaling in cytomegalovirus-infected cells. J Immunol 192: 447–458. 10.4049/jimmunol.1203516 24319264

[46] Krause E, de Graaf M, Fliss PM, Dolken L, Brune W (2014) Murine cytomegalovirus virion-associated protein M45 mediates rapid NF-kappaB activation after infection. J Virol 88: 9963–9975. 10.1128/JVI.00684-14 24942588 PMC4136316

[47] Fliss PM, Jowers TP, Brinkmann MM, Holstermann B, Mack C, et al (2012) Viral mediated redirection of NEMO/IKKgamma to autophagosomes curtails the inflammatory cascade. PLoS Pathog 8: e1002517 10.1371/journal.ppat.1002517 22319449 PMC3271075

[48] Kattenhorn LM, Mills R, Wagner M, Lomsadze A, Makeev V, et al (2004) Identification of proteins associated with murine cytomegalovirus virions. J Virol 78: 11187–11197. 10.1128/JVI.78.20.11187-11197.2004 15452238 PMC521832

[49] Kalejta RF (2008) Tegument proteins of human cytomegalovirus. Microbiol Mol Biol Rev 72: 249–265. 10.1128/MMBR.00040-07 18535146 PMC2415745

[50] Lacaze P, Forster T, Ross A, Kerr LE, Salvo-Chirnside E, et al (2011) Temporal profiling of the coding and noncoding murine cytomegalovirus transcriptomes. J Virol 85: 6065–6076. 10.1128/JVI.02341-10 21471238 PMC3126304

[51] Fossum E, Friedel CC, Rajagopala SV, Titz B, Baiker A, et al (2009) Evolutionarily conserved herpesviral protein interaction networks. PLoS Pathog 5: e1000570 10.1371/journal.ppat.1000570 19730696 PMC2731838

[52] Yoneyama M, Kikuchi M, Natsukawa T, Shinobu N, Imaizumi T, et al (2004) The RNA helicase RIG-I has an essential function in double-stranded RNA-induced innate antiviral responses. Nat Immunol 5: 730–737. 10.1038/ni1087 15208624

[53] Mibayashi M, Martinez-Sobrido L, Loo YM, Cardenas WB, Gale M Jr., et al (2007) Inhibition of retinoic acid-inducible gene I-mediated induction of beta interferon by the NS1 protein of influenza A virus. J Virol 81: 514–524. 10.1128/JVI.01265-06 17079289 PMC1797471

[54] Guo Z, Chen LM, Zeng H, Gomez JA, Plowden J, et al (2007) NS1 protein of influenza A virus inhibits the function of intracytoplasmic pathogen sensor, RIG-I. Am J Respir Cell Mol Biol 36: 263–269. 10.1165/rcmb.2006-0283RC 17053203

[55] Pichlmair A, Schulz O, Tan CP, Naslund TI, Liljestrom P, et al (2006) RIG-I-mediated antiviral responses to single-stranded RNA bearing 5'-phosphates. Science 314: 997–1001. 10.1126/science.1132998 17038589

[56] Opitz B, Rejaibi A, Dauber B, Eckhard J, Vinzing M, et al (2007) IFNbeta induction by influenza A virus is mediated by RIG-I which is regulated by the viral NS1 protein. Cell Microbiol 9: 930–938. 10.1111/j.1462-5822.2006.00841.x 17140406

[57] Gitlin L, Barchet W, Gilfillan S, Cella M, Beutler B, et al (2006) Essential role of mda-5 in type I IFN responses to polyriboinosinic:polyribocytidylic acid and encephalomyocarditis picornavirus. Proc Natl Acad Sci U S A 103: 8459–8464. 10.1073/pnas.0603082103 16714379 PMC1464000

[58] Kato H, Sato S, Yoneyama M, Yamamoto M, Uematsu S, et al (2005) Cell type-specific involvement of RIG-I in antiviral response. Immunity 23: 19–28. 10.1016/j.immuni.2005.04.010 16039576

[59] Gao D, Wu J, Wu YT, Du F, Aroh C, et al (2013) Cyclic GMP-AMP synthase is an innate immune sensor of HIV and other retroviruses. Science 341: 903–906. 10.1126/science.1240933 23929945 PMC3860819

[60] Lahaye X, Satoh T, Gentili M, Cerboni S, Conrad C, et al (2013) The capsids of HIV-1 and HIV-2 determine immune detection of the viral cDNA by the innate sensor cGAS in dendritic cells. Immunity 39: 1132–1142. 10.1016/j.immuni.2013.11.002 24269171

[61] Lam E, Stein S, Falck-Pedersen E (2014) Adenovirus detection by the cGAS/STING/TBK1 DNA sensing cascade. J Virol 88: 974–981. 10.1128/JVI.02702-13 24198409 PMC3911663

[62] Dansako H, Ueda Y, Okumura N, Satoh S, Sugiyama M, et al (2016) The cyclic GMP-AMP synthetase—STING signaling pathway is required for both the innate immune response against HBV and the suppression of HBV assembly. FEBS J 283: 144–156. 10.1111/febs.13563 26471009

[63] Sun C, Schattgen SA, Pisitkun P, Jorgensen JP, Hilterbrand AT, et al (2015) Evasion of innate cytosolic DNA sensing by a gammaherpesvirus facilitates establishment of latent infection. J Immunol 194: 1819–1831. 10.4049/jimmunol.1402495 25595793 PMC4323864

[64] Lio CW, McDonald B, Takahashi M, Dhanwani R, Sharma N, et al (2016) cGAS-STING signaling regulates initial innate control of cytomegalovirus infection. J Virol 90: 7789–7797. 10.1128/JVI.01040-16 27334590 PMC4988162

[65] Hwang S, Kim KS, Flano E, Wu TT, Tong LM, et al (2009) Conserved herpesviral kinase promotes viral persistence by inhibiting the IRF-3-mediated type I interferon response. Cell Host Microbe 5: 166–178. 10.1016/j.chom.2008.12.013 19218087 PMC2749518

[66] Krug A, French AR, Barchet W, Fischer JA, Dzionek A, et al (2004) TLR9-dependent recognition of MCMV by IPC and DC generates coordinated cytokine responses that activate antiviral NK cell function. Immunity 21: 107–119. 10.1016/j.immuni.2004.06.007 15345224

[67] Tabeta K, Hoebe K, Janssen EM, Du X, Georgel P, et al (2006) The Unc93b1 mutation 3d disrupts exogenous antigen presentation and signaling via Toll-like receptors 3, 7 and 9. Nat Immunol 7: 156–164. 10.1038/ni1297 16415873

[68] Delale T, Paquin A, Asselin-Paturel C, Dalod M, Brizard G, et al (2005) MyD88-dependent and -independent murine cytomegalovirus sensing for ifn-α release and initiation of immune responses in vivo. J Immunol 175: 6723–6732. 16272328 10.4049/jimmunol.175.10.6723

[69] Marijanovic Z, Ragimbeau J, Kumar KG, Fuchs SY, Pellegrini S (2006) TYK2 activity promotes ligand-induced IFNAR1 proteolysis. Biochem J 397: 31–38. 10.1042/BJ20060272 16551269 PMC1479745

[70] Fujita T, Miyamoto M, Kimura Y, Hammer J, Taniguchi T (1989) Involvement of a cis-element that binds an H2TF-1/NF kappa B like factor(s) in the virus-induced interferon-beta gene expression. Nucleic Acids Res 17: 3335–3346. 2566973 10.1093/nar/17.9.3335 PMC317778

[71] Yoneyama M, Suhara W, Fukuhara Y, Sato M, Ozato K, et al (1996) Autocrine amplification of type I interferon gene expression mediated by interferon stimulated gene factor 3 (ISGF3). J Biochem 120: 160–169. 8864859 10.1093/oxfordjournals.jbchem.a021379

[72] Juranic Lisnic V, Babic Cac M, Lisnic B, Trsan T, Mefferd A, et al (2013) Dual analysis of the murine cytomegalovirus and host cell transcriptomes reveal new aspects of the virus-host cell interface. PLoS Pathog 9: e1003611 10.1371/journal.ppat.1003611 24086132 PMC3784481

[73] Lienenklaus S, Cornitescu M, Zietara N, Lyszkiewicz M, Gekara N, et al (2009) Novel reporter mouse reveals constitutive and inflammatory expression of IFN-beta in vivo. J Immunol 183: 3229–3236. 10.4049/jimmunol.0804277 19667093

[74] Podlech J, Holtappels R, Pahl-Seibert MF, Steffens HP, Reddehase MJ (2000) Murine model of interstitial cytomegalovirus pneumonia in syngeneic bone marrow transplantation: persistence of protective pulmonary CD8-T-cell infiltrates after clearance of acute infection. J Virol 74: 7496–7507. 10906203 10.1128/jvi.74.16.7496-7507.2000 PMC112270

[75] Böhm V, Podlech J, Thomas D, Deegen P, Pahl-Seibert MF, et al (2008) Epitope-specific in vivo protection against cytomegalovirus disease by CD8 T cells in the murine model of preemptive immunotherapy. Med Microbiol Immunol 197: 135–144. 10.1007/s00430-008-0092-3 18340461

[76] Sacher T, Podlech J, Mohr CA, Jordan S, Ruzsics Z, et al (2008) The major virus-producing cell type during murine cytomegalovirus infection, the hepatocyte, is not the source of virus dissemination in the host. Cell Host Microbe 3: 263–272. 10.1016/j.chom.2008.02.014 18407069

[77] Thomas S, Klobuch S, Podlech J, Plachter B, Hoffmann P, et al (2015) Evaluating human t-cell therapy of cytomegalovirus organ disease in HLA-transgenic mice. PLoS Pathog 11: e1005049 10.1371/journal.ppat.1005049 26181057 PMC4504510

[78] Lemmermann NA, Reddehase MJ (2016) Refining human T-cell immunotherapy of cytomegalovirus disease: a mouse model with 'humanized' antigen presentation as a new preclinical study tool. Med Microbiol Immunol 205: 549–561. 10.1007/s00430-016-0471-0 27539576

[79] Lanfranca MP, Mostafa HH, Davido DJ (2014) HSV-1 ICP0: An E3 Ubiquitin Ligase That Counteracts Host Intrinsic and Innate Immunity. Cells 3: 438–454. 10.3390/cells3020438 24852129 PMC4092860

[80] Tabeta K, Georgel P, Janssen E, Du X, Hoebe K, et al (2004) Toll-like receptors 9 and 3 as essential components of innate immune defense against mouse cytomegalovirus infection. Proc Natl Acad Sci U S A 101: 3516–3521. 10.1073/pnas.0400525101 14993594 PMC373494

[81] Tam A, Zhu J, Hai R, Haghjoo E, Tong T, et al (2003) Murine Cytomegalovirus with a Transposon Insertional Mutation at Open Reading Frame M35 Is Defective in Growth In Vivo. J Virol 77: 7746–7755. 10.1128/JVI.77.14.7746-7755.2003 12829814 PMC161956

[82] Wang B, Nishimura M, Tang H, Kawabata A, Mahmoud NF, et al (2016) Crystal structure of human herpesvirus 6B tegument protein U14. PLoS Pathog 12: e1005594 10.1371/journal.ppat.1005594 27152739 PMC4859480

[83] Salsman J, Jagannathan M, Paladino P, Chan PK, Dellaire G, et al (2012) Proteomic profiling of the human cytomegalovirus ul35 gene products reveals a role for UL35 in the DNA repair response. J Virol 86: 806–820. 10.1128/JVI.05442-11 22072767 PMC3255807

[84] Trigg BJ, Ferguson BJ (2015) Functions of DNA damage machinery in the innate immune response to DNA virus infection. Curr Opin Virol 15: 56–62. 10.1016/j.coviro.2015.08.001 26318640

[85] Hartlova A, Erttmann SF, Raffi FA, Schmalz AM, Resch U, et al (2015) DNA damage primes the type I interferon system via the cytosolic DNA sensor STING to promote anti-microbial innate immunity. Immunity 42: 332–343. 10.1016/j.immuni.2015.01.012 25692705

[86] Chan YK, Gack MU (2016) Viral evasion of intracellular DNA and RNA sensing. Nat Rev Microbiol 14: 360–373. 10.1038/nrmicro.2016.45 27174148 PMC5072394

[87] Ma Z, Damania B (2016) The cGAS-STING Defense Pathway and Its Counteraction by Viruses. Cell Host Microbe 19: 150–158. 10.1016/j.chom.2016.01.010 26867174 PMC4755325

[88] Orzalli MH, Knipe DM (2014) Cellular sensing of viral DNA and viral evasion mechanisms. Annu Rev Microbiol 68: 477–492. 10.1146/annurev-micro-091313-103409 25002095 PMC4348004

[89] Christensen MH, Paludan SR (2017) Viral evasion of DNA-stimulated innate immune responses. Cell Mol Immunol 14: 4–13. 10.1038/cmi.2016.06 26972769 PMC5214947

[90] Jin L, Xu LG, Yang IV, Davidson EJ, Schwartz DA, et al (2011) Identification and characterization of a loss-of-function human MPYS variant. Genes Immun 12: 263–269. 10.1038/gene.2010.75 21248775 PMC3107388

[91] Tischer BK, Smith GA, Osterrieder N (2010) En passant mutagenesis: a two step markerless red recombination system. Methods Mol Biol 634: 421–430. 10.1007/978-1-60761-652-8_30 20677001

[92] Jordan S, Krause J, Prager A, Mitrovic M, Jonjic S, et al (2011) Virus progeny of murine cytomegalovirus bacterial artificial chromosome pSM3fr show reduced growth in salivary Glands due to a fixed mutation of MCK-2. J Virol 85: 10346–10353. 10.1128/JVI.00545-11 21813614 PMC3196435

[93] Erlandsson L, Blumenthal R, Eloranta ML, Engel H, Alm G, et al (1998) Interferon-beta is required for interferon-alpha production in mouse fibroblasts. Curr Biol 8: 223–226. 9501984 10.1016/s0960-9822(98)70086-7

[94] Trotard M, Tsopoulidis N, Tibroni N, Willemsen J, Binder M, et al (2015) Sensing of HIV-1 infection in Tzm-bl cells with reconstituted expression of STING. J Virol 90: 2064–2076. 10.1128/JVI.02966-15 26656698 PMC4733976

[95] Munks MW, Gold MC, Zajac AL, Doom CM, Morello CS, et al (2006) Genome-wide analysis reveals a highly diverse CD8 T cell response to murine cytomegalovirus. J Immunol 176: 3760–3766. 16517745 10.4049/jimmunol.176.6.3760

[96] Bussey KA, Reimer E, Todt H, Denker B, Gallo A, et al (2014) The gammaherpesviruses Kaposi's sarcoma-associated herpesvirus and murine gammaherpesvirus 68 modulate the Toll-like receptor-induced proinflammatory cytokine response. J Virol 88: 9245–9259. 10.1128/JVI.00841-14 24899179 PMC4136288

[97] Sander G, Konrad A, Thurau M, Wies E, Leubert R, et al (2008) Intracellular localization map of human herpesvirus 8 proteins. J Virol 82: 1908–1922. 10.1128/JVI.01716-07 18077714 PMC2258699

[98] Bialas KM, Desmet EA, Takimoto T (2012) Specific residues in the 2009 H1N1 swine-origin influenza matrix protein influence virion morphology and efficiency of viral spread in vitro. PLoS ONE 7: e50595 10.1371/journal.pone.0050595 23209789 PMC3507794

[99] Basler CF, Reid AH, Dybing JK, Janczewski TA, Fanning TG, et al (2001) Sequence of the 1918 pandemic influenza virus nonstructural gene (NS) segment and characterization of recombinant viruses bearing the 1918 NS genes. Proc Natl Acad Sci U S A 98: 2746–2751. 10.1073/pnas.031575198 11226311 PMC30210

[100] Labzin LI, Schmidt SV, Masters SL, Beyer M, Krebs W, et al (2015) ATF3 Is a Key Regulator of Macrophage IFN Responses. J Immunol 195: 4446–4455. 10.4049/jimmunol.1500204 26416280

[101] Shi GP, Villadangos JA, Dranoff G, Small C, Gu L, et al (1999) Cathepsin S required for normal MHC class II peptide loading and germinal center development. Immunity 10: 197–206. 10072072 10.1016/s1074-7613(00)80020-5

[102] Yokoyama WM, Christensen M, Santos GD, Miller D, Ho J, et al (2013) Production of monoclonal antibodies. Curr Protoc Immunol:102:Unit 2.5 10.1002/0471142735.im0205s102 24510488

[103] Lemmermann NA, Podlech J, Seckert CK, Kropp KA, Grzimek NK, et al (2010) CD8 T-cell immunotherapy of cytomegalovirus disease in the murine model In: Kabelitz D, Kaufmann SHE, editors. Methods in Microbiology: Immunology of Infection. 3rd ed Academic Press, London pp 369–420.

[104] Podlech J, Holtappels R, Grzimek NK, Reddehase MJ (2002) Animal models: murine cytomegalovirus In: Kaufmann SHE, Kabelitz D, editors. Methods in Microbiology: Immunology of Infection. 2nd ed Academic Press, London Pp 493–525.

[105] Suzuki K, Bose P, Leong-Quong RY, Fujita DJ, Riabowol K (2010) REAP: A two minute cell fractionation method. BMC Research Notes 3: 294 10.1186/1756-0500-3-294 21067583 PMC2993727

