# Supplementary figures and images for: The murine cytomegalovirus M35 protein antagonizes type I IFN induction downstream of pattern recognition receptors by targeting NF-κB mediated transcription
Source: PLoS Pathog. 2017 May 25;13(5):e1006382. doi: 10.1371/journal.ppat.1006382 (PMC5444856; doi:10.1371/journal.ppat.1006382)

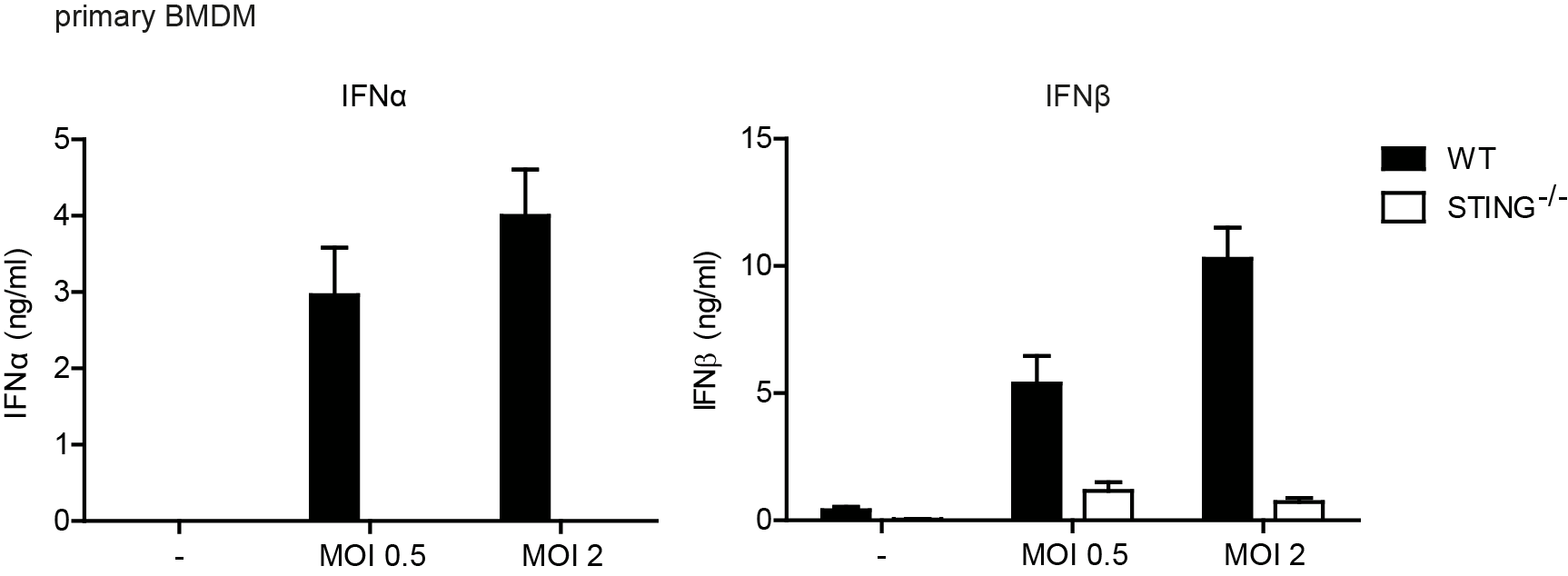

Supplement: S1 Fig — Primary BMDM generated from wildtype (WT) and STING knockout mice were infected with MCMV-GFP at MOI 0.5 or 2 or left uninfected and IFNα/β levels at 16 hours p.i. were analyzed by ELISA. Data is shown as mean ± SD of three independent experiments. (TIF) [file ppat.1006382.s001.tif]

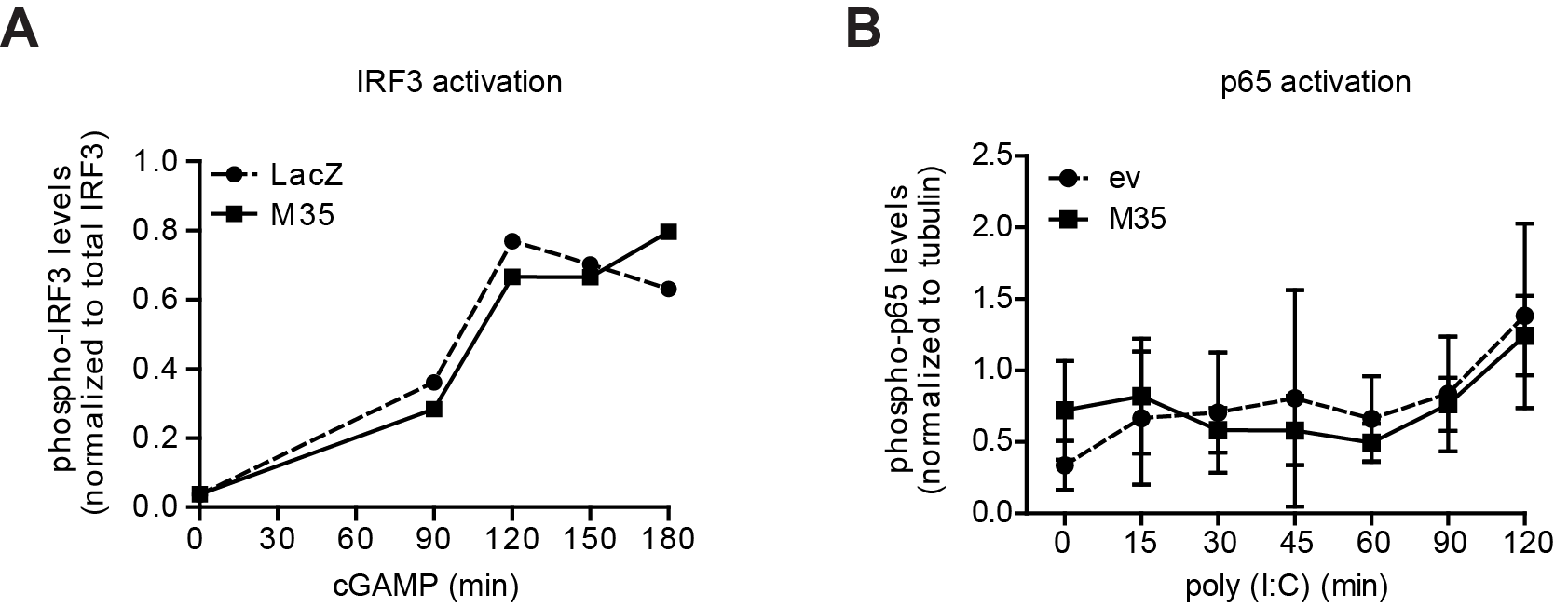

Supplement: S2 Fig — (A) Quantification of phospho-IRF3 levels relative to total IRF3 levels was performed on Fig 4A using ImageJ. (B) Quantification of phospho-p65 levels relative to total tubulin levels was performed on three independent experiments using ImageJ. One representative immunoblot is shown in Fig 4C. (TIF) [file ppat.1006382.s002.tif]

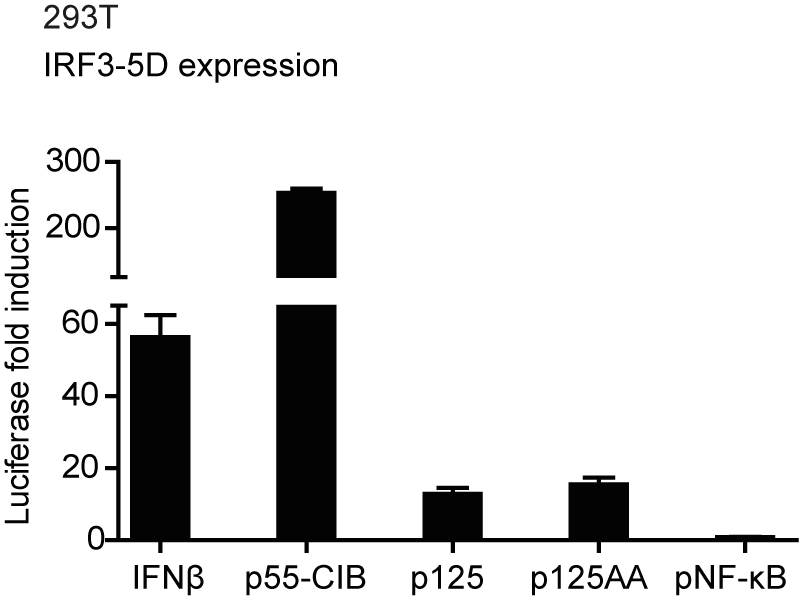

Supplement: S3 Fig — 293T cells were co-transfected with expression plasmids for either the constitutively active form of IRF3 designated IRF3-5D (stimulated) or GFP (unstimulated) together with the pRL-TK luciferase plasmid, pcDNA and the IFNβ, p55-CIB, pPRD-III/I, p125, p125AA or pNF-κB luciferase plasmids. At 20 hours post transfection, cells were lysed and luciferase production was analyzed. Luciferase fold induction was calculated based on firefly luciferase values normalized to Renilla luciferase from stimulated samples divided by corresponding values from unstimulated samples. Data set is combined from two independent experiments and represented as mean ± SD. (TIF) [file ppat.1006382.s003.tif]

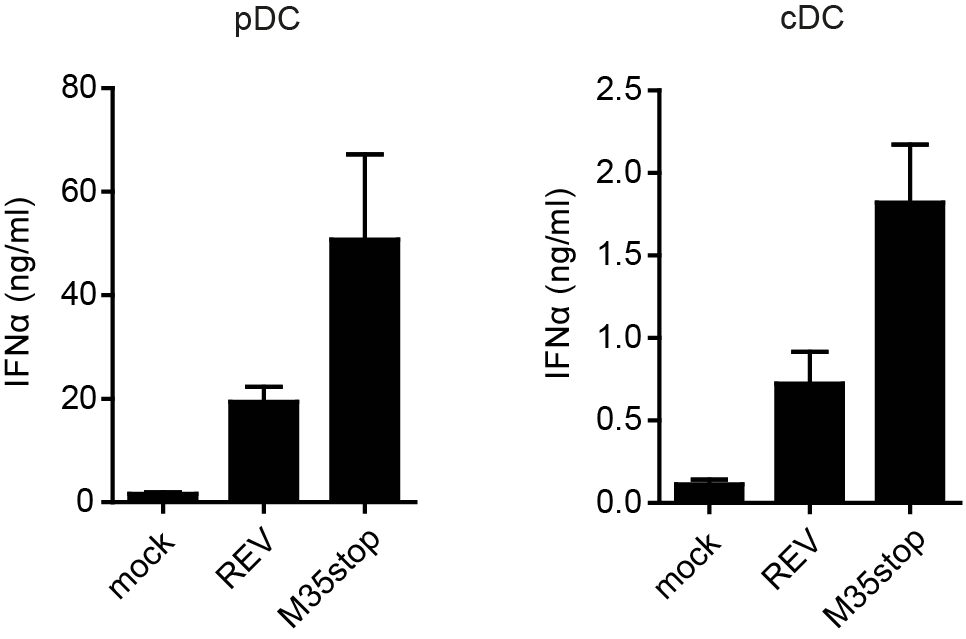

Supplement: S4 Fig — pDC and cDC were infected with MCMV-M35stop-REV (REV) or MCMV-M35stop (M35stop) at an MOI of 0.01 (pDC), 0.1 (cDC), or left uninfected (mock). Supernatants were harvested 16 hours p.i. for quantification of IFNα levels by ELISA. Data is shown as mean ± SD and representative of three independent experiments. (TIF) [file ppat.1006382.s004.tif]

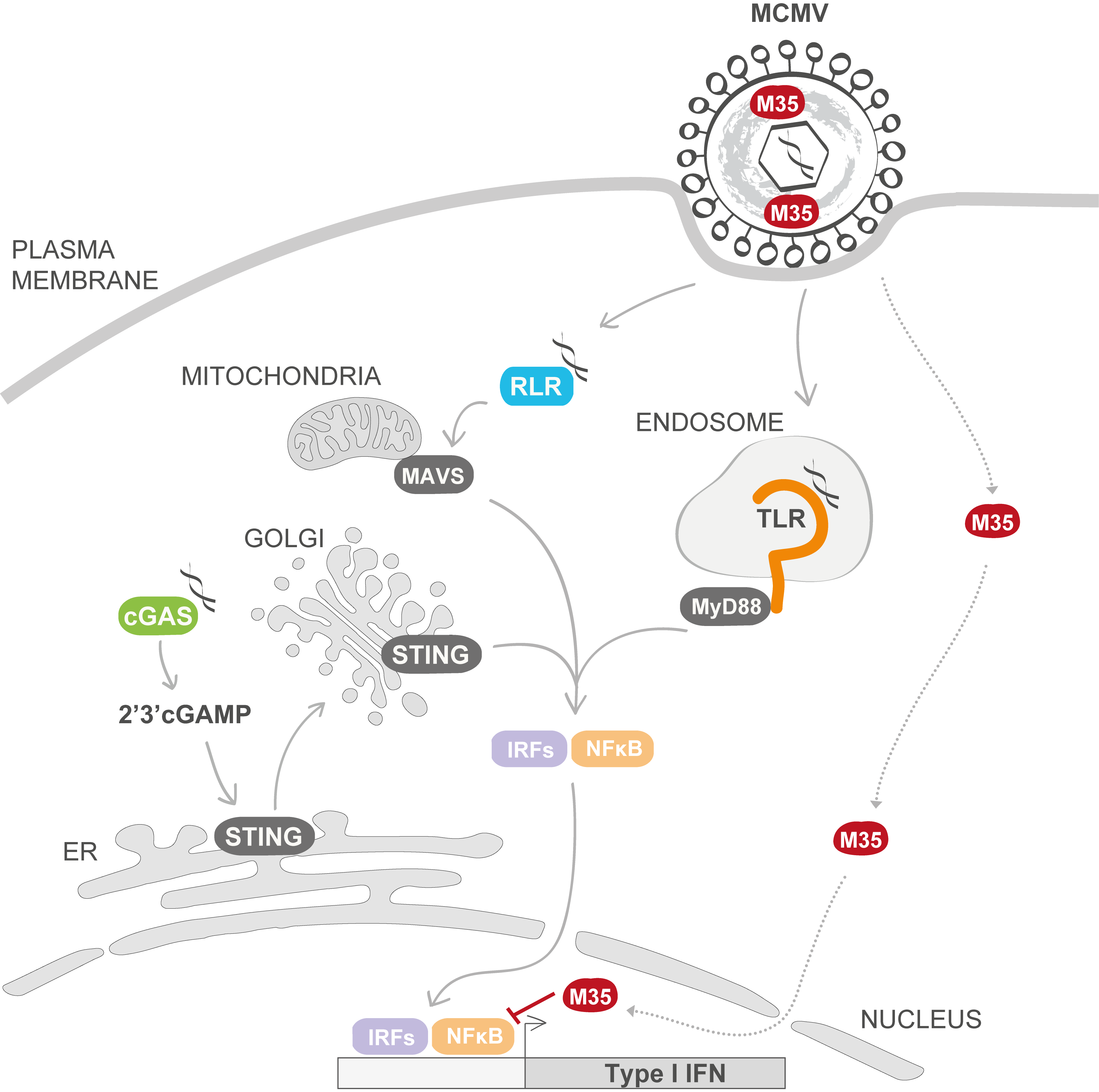

Supplement: S5 Fig — Sensing of MCMV infection by multiple PRR, including cGAS, RIG-I-like receptors (RLR), and Toll-like receptors (TLR), activates signaling cascades leading to the production of antiviral type I IFN. Upon MCMV infection, tegument M35 is rapidly transported to the nucleus in order to specifically interfere with NF-κB-mediated type I IFN transcription. (TIF) [file ppat.1006382.s005.tif]
